# Expanding the substrate spectrum in engineered *Pseudomonas taiwanensis* for efficient production of 4-coumarate from lignocellulosic sugars

**DOI:** 10.1186/s12934-026-03014-w

**Published:** 2026-05-23

**Authors:** Benedikt Wynands, Sophia Feltes, Nadine Teófilo da Silva, Tino Polen, Nick Wierckx

**Affiliations:** Institute of Bio- and Geosciences, IBG-1: Biotechnology, Forschungszentrum Jülich GmbH, 52425 Jülich, Germany

**Keywords:** Aromatics, Biocatalysis, 4-Coumarate, Metabolic engineering, *Pseudomonas*, Xylose, Arabinose, Tyrosine ammonia-lyase

## Abstract

Aromatics are important building blocks for polymers, pharmaceuticals, and advanced materials, but their current production relies on petrochemical processes. Biotechnological de novo production from renewable bio-based feedstocks with microbial cell factories provides a sustainable alternative. In this study, we enhanced 4-coumarate production in *Pseudomonas taiwanensis* from glucose and glycerol compared to previously published producers. This was achieved through heterologous expression of tyrosine ammonia-lyase (TAL) from *Rivularia* sp. PCC7116, which debottlenecked the specific deamination of tyrosine. Moreover, deletion of the phosphoenolpyruvate carboxylase-encoding gene *ppc* further increased the production. Subsequently, the substrate spectrum for efficient aromatics production was expanded to include the abundant pentoses, xylose and arabinose. Heterologous non-oxidative assimilation pathways were integrated into *P. taiwanensis* GRC3 *chassis* strains and growth on xylose and arabinose was improved through adaptive laboratory evolution, whole-genome sequencing, and reverse engineering. Optimized catabolic modules were then transferred to producer strains to enhance or enable 4-coumarate production from xylose and arabinose. Notably, the product yield on xylose increased approximately 3.5-fold with the non-oxidative xylose isomerase pathway compared to the oxidative native Weimberg pathway, without compromising yields on glucose. For the final strain, *P. taiwanensis* GRC3Δ6-TYR2Δ*ppc*-REXA-*attTn7*::*P*_*14f*_*-RpcTAL*, product yields were significantly higher on xylose (38.2% (Cmol/Cmol)) and arabinose (39.7% (Cmol/Cmol)) than on glucose (26.0% (Cmol/Cmol)). 4-Coumarate production was characterized on mixtures of glucose, xylose, and arabinose to mimic lignocellulosic hydrolysate feedstocks, with the best reverse-engineered xylose- and arabinose-metabolizing 4-coumarate producer significantly outperforming the reference strain.

**Supplementary Information:**

The online version contains supplementary material available at 10.1186/s12934-026-03014-w.

## Background

Aromatic chemicals are indispensable in modern society, with applications across a wide range of industrial sectors. Their conventional production relies on petrochemical processes that are energy-intensive, polluting, and greenhouse gas-emitting. For environmental and sustainability reasons, there is an urgent need to transition from the traditional manufacturing of aromatics to more environmentally friendly processes. In this context, the biotechnological de novo production of aromatic chemicals from renewable substrates using microbes as whole-cell biocatalysts is a promising approach. This research field is extensively investigated in academia [1] and first large-scale processes, such as bio-based aniline production, are being developed in industry [2, 3]. In previous studies, we established *Pseudomonas taiwanensis* VLB120 and its genome-reduced *chassis* strains as cell factories for the bioproduction of a wide range of aromatics [4–8]. In Wynands et al. [9], we developed a platform strain for the production of 4-coumarate from glucose or glycerol by applying an unspecific phenylalanine/tyrosine ammonia-lyase (*Rt*PAL). Additional strain engineering was required to reduce the abundance of phenylalanine and thereby limit *trans*-cinnamate accumulation. Although high 4-coumarate production was achieved, the obtained strain showed reduced growth rates and an overall decreased phenylpropanoid biosynthesis [9]. Thus, debottlenecking specific tyrosine deamination by superior tyrosine ammonia-lyase (TAL) enzymes remained an important objective for further strain development efforts.

**Fig. 1:**
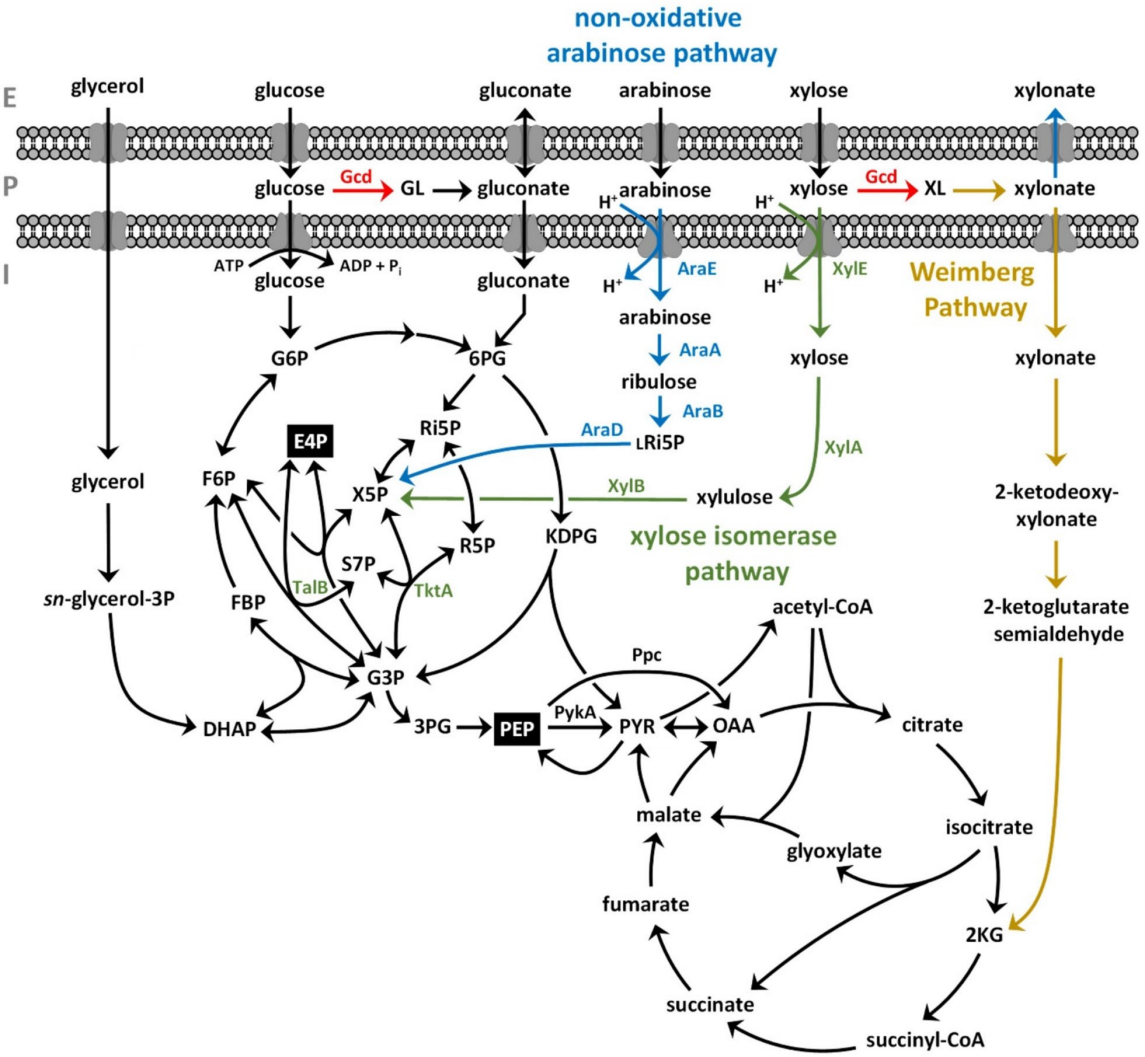
Schematic illustration of the central carbon metabolism of *P. taiwanensis* including heterologous pathways and enzymes for pentose metabolism. The native Weimberg pathway is indicated by golden yellow arrows. The membrane-bound periplasmic glucose dehydrogenase (Gcd) was deleted to disrupt this native xylose assimilation pathway (shown in red). The heterologous xylose isomerase pathway is illustrated in green, the heterologous non-oxidative arabinose pathway in blue. Transaldolase (TalB) and transketolase (TktA) were co-expressed with the xylose isomerase module (also shown in green). Precursors of the shikimate pathway, i.e., PEP and E4P, are highlighted in black boxes. E, extracellular; P, periplasm; I, intracellular; G6P, glucose 6-phosphate; GL, gluconolactone; 6PG, 6-phosphogluconate; F6P, fructose 6-phosphate; FBP, fructose 1,6-bisphosphate; G3P, glyceraldehyde 3-phosphate; 3PG, 3-phosphoglycerate; DHAP, dihydroxyacetone phosphate; PEP, phosphoenolpyruvate; PYR, pyruvate; KDPG, 2-keto-3-deoxy-6-phosphogluconate; Ri5P, ribulose 5-phosphate; lRi5P, l-ribulose 5-phosphate; R5P, ribose 5-phosphate; X5P, xylulose 5-phosphate; E4P, erythrose 4-phosphate; S7P, sedoheptulose 7-phosphate; XL, xylonolactone; OAA, oxaloacetate; 2 KG, 2-ketoglutarate; PykA, pyruvate kinase A; Ppc, phosphoenolpyruvate carboxylase

Pseudomonads are promising workhorses for industrial biotechnology due to their genetic tractability and inherent resilience [10], and have thus been extensively applied in aromatics biocatalysis approaches [11]. Although they generally possess a versatile metabolism, many *Pseudomonas* strains currently applied in the field of industrial biotechnology are either unable to catabolize abundant pentose sugars or they are equipped with catabolic pathways that are suboptimal for product formation. For this reason, the application of agricultural side streams or lignocellulosic biomass as second-generation (2G) feedstocks is limited when using *Pseudomonas* cell factories. Yet, the integration of 2G feedstocks into biotechnological production processes is crucial to avoid competition with food and feed. Furthermore, feedstock prices are a key-determining factor of production costs and the lower cost of 2G feedstocks can help to achieve cost competitiveness. While d-glucose is generally metabolized, the assimilation of other abundant lignocellulosic sugars, such as d-xylose and l-arabinose, is not widespread in biotechnologically relevant *Pseudomonas* strains. This shortcoming was addressed in several metabolic engineering approaches through implementation of heterologous pathways to enable growth of *P. putida* on the abovementioned sugars [12–20]. In contrast to *P. putida* KT2440, *P. taiwanensis* VLB120 is natively equipped with the Weimberg pathway (Fig. 1) and thus able to assimilate xylose via α-ketoglutarate as central metabolite [21]. In general, this is most favorable for the bioproduction of TCA cycle-derived molecules. Recently, metabolic bottlenecks within the Weimberg pathway of *P. taiwanensis* VLB120 were identified and addressed through genetic engineering to enhance oxidative xylose utilization [22]. However, in the context of aromatics de novo biosynthesis the non-oxidative xylose isomerase pathway is considered more promising due to a potentially enhanced provision of erythrose 4-phosphate (E4P) as a precursor [16]. Accordingly, this pathway was shown to be beneficial in *P. putida* S12 and *P. putida* KT2440 for the production of aromatics [16, 23] and shikimate pathway-derived *cis*,*cis*-muconate [24] and β-ketoadipate [25].

In the present study, we further enhanced 4-coumarate production from glucose and glycerol through heterologous expression of the recently identified *Rpc*TAL [26] in tyrosine-overproducing *P. taiwanensis* GRC3Δ5-TYR2 and the deletion of phosphoenolpyruvate carboxylase-encoding gene (Δ*ppc*). Furthermore, we rearranged the metabolic wiring from oxidative to non-oxidative xylose assimilation applying the xylose isomerase pathway in genome-reduced *chassis* strain *P. taiwanensis* GRC3 and enabled non-oxidative utilization of arabinose to channel those sugars into the pentose phosphate pathway (Fig. 1). Upon heterologous pathway integration, the assimilation of xylose and arabinose was improved through adaptive laboratory evolution and reverse engineering of identified mutations. Optimized pathway modules were then applied in producer strains to improve or enable 4-coumarate biosynthesis from these sugars. Ultimately, 4-coumarate production was demonstrated from mock lignocellulosic hydrolysate medium containing glucose, xylose, and arabinose.

## Results and discussion

### Enhanced 4-coumarate production through heterologous expression of *Rpc*TAL

**Fig. 2:**
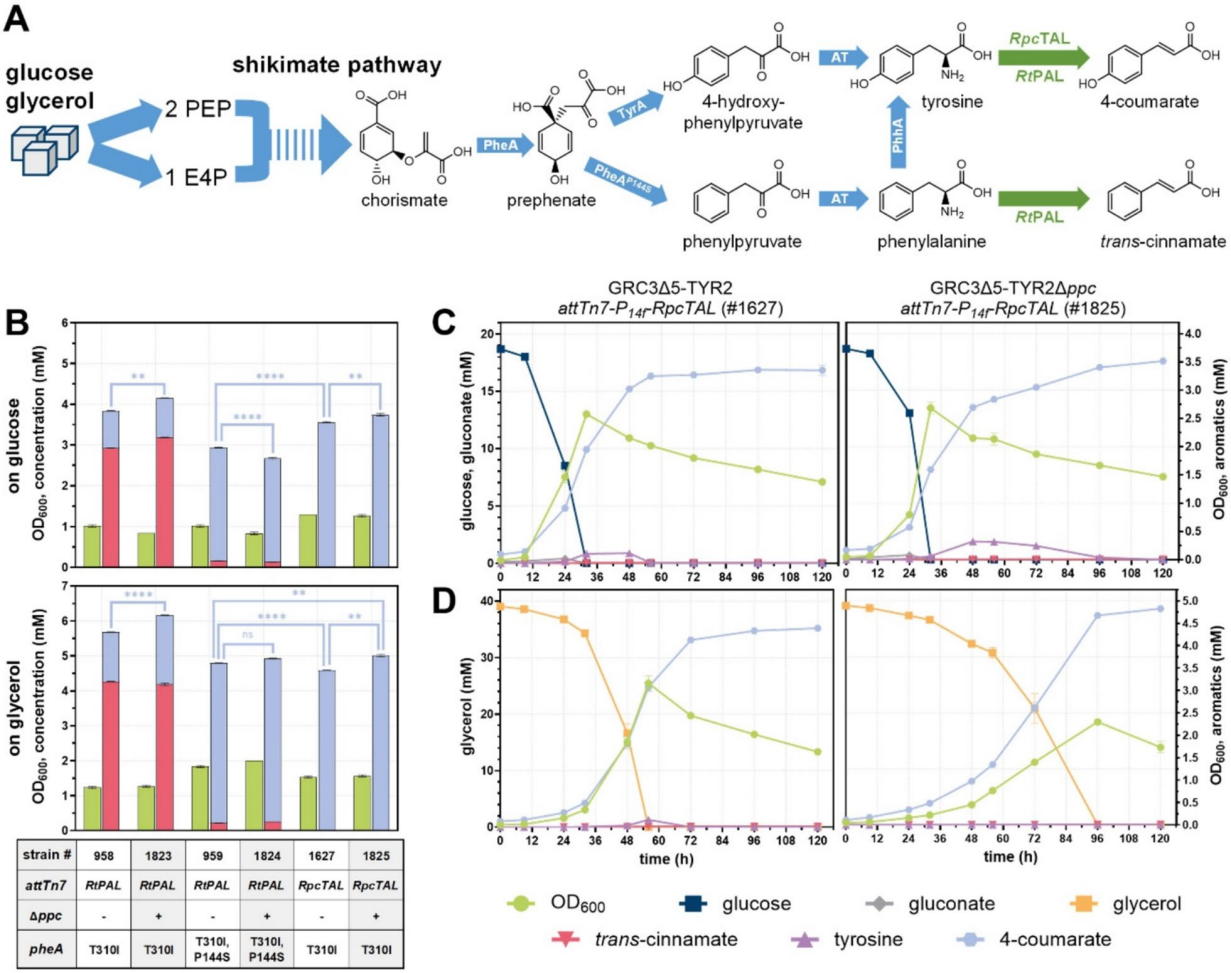
Biosynthetic pathway for the production of 4-coumarate, applying the unspecific *Rt*PAL and tyrosine-specific *Rpc*TAL (**A**). Due to *Rt*PAL’s affinity for phenylalanine, this ammonia-lyase was previously combined with the PheA^P144S^ modification to reduce phenylalanine abundance and thus *trans*-cinnamate formation. Blue arrows indicate native enzymes, green arrows represent heterologously expressed enzymes. 4-Coumarate production was assessed from 20 mM glucose or 40 mM glycerol in small-scale 24-well plate cultivations after 120 h (**B**) and shake flask cultivations from 20 mM glucose (**C**) or 40 mM glycerol (**D**) in twofold-buffered mineral salt medium (MSM) using the indicated strains. Mean values were calculated from a dataset of three biological replicates (*n* = 3). Error bars represent the standard deviation. Statistical significance was evaluated by *t*-tests with *p* < 0.01 (**), *p* < 0.0001 (****), or not significant (ns). PheA, bifunctional chorismate mutase/prephenate dehydratase; TyrA, bifunctional 3-phosphoshikimate 1-carboxyvinyltransferase/prephenate dehydrogenase; AT, aminotransferases; PhhA, phenylalanine 4-hydroxylase; *Rt*PAL, unspecific phenylalanine ammonia-lyase from *Rhodosporidium toruloides*; *Rpc*TAL, tyrosine ammonia-lyase from *Rivularia* sp. PCC7116

In our previous study, we engineered tyrosine-overproducing *P. taiwanensis* for efficient 4-coumarate production from glucose and glycerol. Best key performance indicators were achieved with an unspecific phenylalanine ammonia-lyase from *Rhodosporidium toruloides* (*Rt*PAL), that deaminates both phenylalanine and tyrosine. To limit the formation of *trans*-cinnamate as a byproduct, the upstream pathway was engineered to reduce the abundance of phenylalanine as a substrate for *Rt*PAL through the introduction of a point mutation (P144S) in the prephenate dehydratase domain of PheA (Fig. 2A). However, this modification could not prevent the formation of *trans*-cinnamate entirely, and growth and overall phenylpropanoid (*trans*-cinnamate + 4-coumarate) production was reduced compared to a strain lacking the *pheA*^P144S^ mutation. With the intention to debottleneck specific tyrosine deamination and further enhance the efficiency of 4-coumarate production, a tyrosine ammonia-lyase from *Rivularia* sp. PCC7116 (*Rpc*TAL) was selected for in vivo testing in *P. taiwanensis* (Fig. 2A) due to its promising in vitro performance [26]. For this, *RpcTAL* was codon-optimized and cloned into the pBG14f_FRT_Kan backbone [27] to allow chromosomal integration into the *attTn7* site of the tyrosine-producing strain GRC3Δ5-TYR2, enabling direct comparison to previously screened combinations of ammonia-lyases and strain backgrounds. In Op de Hipt et al. [28], we already demonstrated that *Rpc*TAL distinctly outperformed *Fj*TAL and *Sts*TAL, which were the best two TALs screened before [9]. In this study, the *Rpc*TAL-expressing strain was compared to the previously engineered GRC3Δ5-TYR2-*attTn7*::*P*_*14f*_*-RtPAL* (without *pheA*^P144S^) and GRC3Δ5-TYR3-*attTn7*::*P*_*14f*_*-RtPAL* (with *pheA*^P144S^). To this end, the respective strains were grown in 24-well microtiter plates with mineral salt medium (MSM), containing either 20 mM glucose or 40 mM glycerol, for 120 h (Fig. 2B). As reported before, the *pheA*^P144S^ mutation was crucial to enhance 4-coumarate production and its selectivity when the *Rt*PAL enzyme was applied (compare #958 to #959). On glucose, the previously engineered producer GRC3Δ5-TYR3-*attTn7*::*P*_*14f*_*-RtPAL* (#959) showed de novo production of 2.77 ± 0.01 mM 4-coumarate with no residual tyrosine and 0.16 ± 0.00 mM *trans*-cinnamate. These concentrations are similar to those reported previously in Wynands et al. [9] for the same strain under identical culture conditions (4-coumarate: 2.64 mM; tyrosine: 0.00 mM; *trans*-cinnamate: 0.11 mM). The newly engineered GRC3Δ5-TYR2 with *attTn7*::*P*_*14f*_*-RpcTAL* (#1627) performed markedly better, producing 3.55 ± 0.01 mM 4-coumarate with no residual tyrosine and only trace amounts of *trans*-cinnamate (<8 µM). Therefore, 4-coumarate de novo production was not only significantly enhanced titer-wise but also regarding the specificity compared to GRC3Δ5-TYR3-*attTn7*::*P*_*14f*_*-RtPAL* (#959) (99.8 vs. 94.6% with *p* < 0.0001).

Phosphoenolpyruvate (PEP) and E4P are the precursors for shikimate pathway-derived aromatics. In order to increase the supply of PEP, pyruvate kinase A was already deleted (Δ*pykA*) previously [4, 9]. Here, we additionally deleted the PEP carboxylase-encoding gene *ppc* to assess whether this could further increase PEP availability for aromatics production, as deletion of *ppc* has previously been shown to enhance the production of shikimate pathway-derived compounds [29]. In the background of GRC3Δ5-TYR2 (without *pheA*^P144S^), the deletion of *ppc* enhanced production of 4-coumarate and overall phenylpropanoids (4-coumarate + *trans*-cinnamate) when *Rt*PAL was expressed (#1823) and that of 4-coumarate when *Rpc*TAL was expressed (#1825). As a consequence, strain GRC3Δ5-TYR2Δ*ppc*-*attTn7*::*P*_*14f*_*-RpcTAL* (#1825) showed the highest production, with a titer of 3.73 ± 0.03 mM 4-coumarate from 20 mM glucose. Interestingly, Δ*ppc* decreased 4-coumarate production in GRC3Δ5-TYR3 expressing the *Rt*PAL (#1824) to only 2.55 ± 0.02 mM, indicating an interplay between the phenylalanine bradytrophy (caused by PheA^P144S^) and the central metabolism. This demonstrates the advantage of *Rpc*TAL, which not only increased specific 4-coumarate production per se, but also reduced metabolic burden to allow for further modifications in the primary metabolism.

On 40 mM glycerol as sole carbon source, similar trends were observed for the respective strains, but overall higher product titers were achieved (Fig. 2B)—a phenomenon that was consistently observed in our previous studies [4, 9]. However, it is noteworthy that 4-coumarate production on glycerol was virtually identical for GRC3Δ5-TYR3-*attTn7*::*P*_*14f*_*-RtPAL* (#959) and GRC3Δ5-TYR2-*attTn7*::*P*_*14f*_*-RpcTAL* (#1627) (4.58 ± 0.01 vs. 4.57 ± 0.00 mM). Yet, the *Rpc*TAL-expressing strain still benefited from a reduced *trans*-cinnamate formation (0.01 ± 0.00 vs. 0.22 ± 0.00 mM). The highest 4-coumarate titer was again achieved by the *Rpc*TAL-expressing strain that harbors the Δ*ppc* modification (#1825), enabling a production of 5.00 ± 0.03 mM.

For a more detailed characterization of growth, production, and substrate consumption, the *Rpc*TAL-expressing strains with (#1825) and without (#1627) the Δ*ppc* modification were cultured in shake flasks, with sampling over time, using either 20 mM glucose (Fig. 2C) or 40 mM glycerol (Fig. 2D) as sole carbon sources.

When glucose served as sole carbon source, both strains showed similar trends (Table 1), although GRC3Δ5-TYR2-*attTn7*::*P*_*14f*_*-RpcTAL* grew slightly faster (estimated *µ* = 0.18 ± 0.00 h^−1^) and consumed glucose quicker than the equivalent Δ*ppc* strain (estimated *µ* = 0.16 ± 0.00 h^−1^) (Fig. 2C). The final product concentration was higher for the *ppc* knockout strain (3.52 ± 0.02 vs. 3.37 ± 0.09 mM), although with no sufficient statistical significance (*p* = 0.099), whereas the difference was significant in the previous 24-well plate screening (*p* < 0.01). Interestingly, a transient tyrosine accumulation was observed for both strains during the stationary phase, indicating that tyrosine deamination is still at least a minor bottleneck when glucose is applied as a carbon source. The highest tyrosine accumulation was observed after 48 h with 0.18 ± 0.02 mM for GRC3Δ5-TYR2-*attTn7*::*P*_*14f*_*-RpcTAL* and 0.33 ± 0.01 for GRC3Δ5-TYR2Δ*ppc*-*attTn7*::*P*_*14f*_*-RpcTAL*. For the latter strain, the accumulation was not only higher, but the full conversion of tyrosine took also significantly longer, associated with a constant increase of 4-coumarate during stationary phase. Of note, almost no *trans*-cinnamate was detected throughout the cultivations (<6 µM), highlighting the in vivo specificity of *Rpc*TAL.

**Table 1:** Key performance indicators for the production of 4-coumarate from glucose or glycerol for *Rpc*TAL-expressing strains

Strain	Carbon source	Growth rate (h^−1^)	Max. OD_600_	4-Coumarate titer (mM)	Volumetric productivity^a^ (mM h^−1^)	Yield (%)^b^	Fig
GRC3Δ5-TYR2-*attTn7*::*P*_*14f*_*-RpcTAL* (#1627)	20 mM glucose	0.18 ± 0.00 (9–24 h)	2.60 ± 0.00 (32 h)	3.37 ± 0.09 (120 h)	0.060 ± 0.000 (0–48 h)	26.8 ± 0.7 (120 h)	2C
GRC3Δ5-TYR2Δ*ppc*-*attTn7*::*P*_*14f*_*-RpcTAL* (#1825)	20 mM glucose	0.16 ± 0.00 (9–32 h)	2.68 ± 0.10 (32 h)	3.52 ± 0.02 (120 h)	0.053 ± 0.001 (0–48 h)	28.2 ± 0.1 (120 h)	2C
GRC3Δ5-TYR2-*attTn7*::*P*_*14f*_*-RpcTAL* (#1627)	40 mM glycerol	0.09 ± 0.00 (24–48 h)	3.18 ± 0.16 (56 h)	4.40 ± 0.01 (120 h)	0.056 ± 0.000 (0–72 h)	33.8 ± 0.1 (120 h)	2D
GRC3Δ5-TYR2Δ*ppc*-*attTn7*::*P*_*14f*_*-RpcTAL* (#1825)	40 mM glycerol	0.05 ± 0.00 (32–56 h)	2.30 ± 0.05 (96 h)	4.83 ± 0.04 (120 h)	0.048 ± 0.000 (0–96 h)	37.0 ± 0.2 (120 h)	2D

^a^Maximum average volumetric productivities were determined over the specified time intervals from the start of the cultivation until production leveled off

^b^Yield is given as carbon-molar percentage (% (Cmol/Cmol))

When glycerol was used as the sole carbon source, the reduced growth performance of GRC3Δ5-TYR2Δ*ppc*-*attTn7*::*P*_*14f*_*-RpcTAL* (*µ* = 0.05 ± 0.00 h^−1^) compared to the strain still possessing *ppc* (*µ* = 0.09 ± 0.00 h^−1^) was much more pronounced (Fig. 2D). This is likely associated to a reduced Entner-Doudoroff pathway flux on glycerol as compared to on glucose [30], and thus an increased impact of the *ppc* deletion especially in the background of Δ*pykA*. While GRC3Δ5-TYR2-*attTn7*::*P*_*14f*_*-RpcTAL* reached its maximum OD_600_ (3.18 ± 0.16) and consumed nearly all glycerol after 56 h, the Δ*ppc* strain reached its highest OD_600_ (2.30 ± 0.05) only after approximately 96 h when glycerol was fully consumed. Hence, the production rate was decreased, but other performance indicators were significantly improved (*p* < 0.01) for GRC3Δ5-TYR2Δ*ppc*-*attTn7*::*P*_*14f*_*-RpcTAL* with a titer of 4.83 ± 0.04 mM and yield of 37.0 ± 0.2% (Cmol/Cmol) (Table 1). A minor transient accumulation of tyrosine was only observed for GRC3Δ5-TYR2-*attTn7*::*P*_*14f*_*-RpcTAL* with 0.16 ± 0.06 mM after 56 h. This indicates that the bottleneck of tyrosine deamination is alleviated when the anabolic flux towards tyrosine is decreased due to lower growth rates. Again, only low *trans*-cinnamate concentrations accumulated (<12 µM).

Overall, the application of *Rpc*TAL enabled further improvements in the production of 4-coumarate on glucose and glycerol compared to previously engineered strains expressing the *Rt*PAL.

### Pathway engineering for assimilation of xylose and arabinose

In multiple previous studies the implementation of the phosphorylative xylose isomerase pathway was already demonstrated in *P. putida* through heterologous expression of xylose isomerase XylA and xylulose kinase XylB from *E. coli*. While in some studies, the expression of these pathway enzymes alone was reported to enable sufficient growth on xylose [14, 17], other studies relied on subsequent adaptive laboratory evolution (ALE) [12], additional rational strain engineering [15], or a combination of both [18, 31, 32] to improve growth performance. Although the Weimberg pathway is absent in *P. putida* KT2440, the deletion of the periplasmic glucose dehydrogenase (encoded by *gcd*) is required to prevent the formation of xylonate as a dead-end product [15, 16]. The additional expression of xylose/H^+^ symporter XylE was shown to debottleneck the uptake of xylose and thus enhance growth [15, 18]. Moreover, the transaldolase (TalB) and transketolase (TktA) were co-expressed to increase the natively low pentose phosphate pathway flux in *P. putida*, which was also shown to significantly improve growth on xylose [18].

In this study, we intended to integrate the xylose isomerase pathway into *P. taiwanensis* to ultimately apply this pathway for 4-coumarate production as the native Weimberg pathway was predicted to be suboptimal for aromatics production due to a lower provision of shikimate pathway precursors. However, we decided to establish and optimize the xylose isomerase pathway first in the *P. taiwanensis* GRC3 *chassis* strain to evaluate the growth performance in a strain without an engineered carbon flux towards tyrosine that competes with growth velocities and overall biomass formation. Thus, the Weimberg pathway was interrupted in *P. taiwanensis* GRC3 through the deletion of *gcd* and subsequently a genetic construct comprising *xylE*, *xylA*, *xylB*, *talB*, and *tktA* was integrated into the intergenic locus of PVLB_22010/15 (Figure S1A). The xylose isomerase pathway module was kindly provided by Elmore et al. [18]. The expression of the *xylAB-talB-tktA* operon is driven by constitutive *P*_*tac*_ promoter, while the divergently expressed *xylE* gene is controlled by *P*_xylE_*, that resulted from an ALE on xylose and increased *xylE* expression [18].

**Fig. 3:**
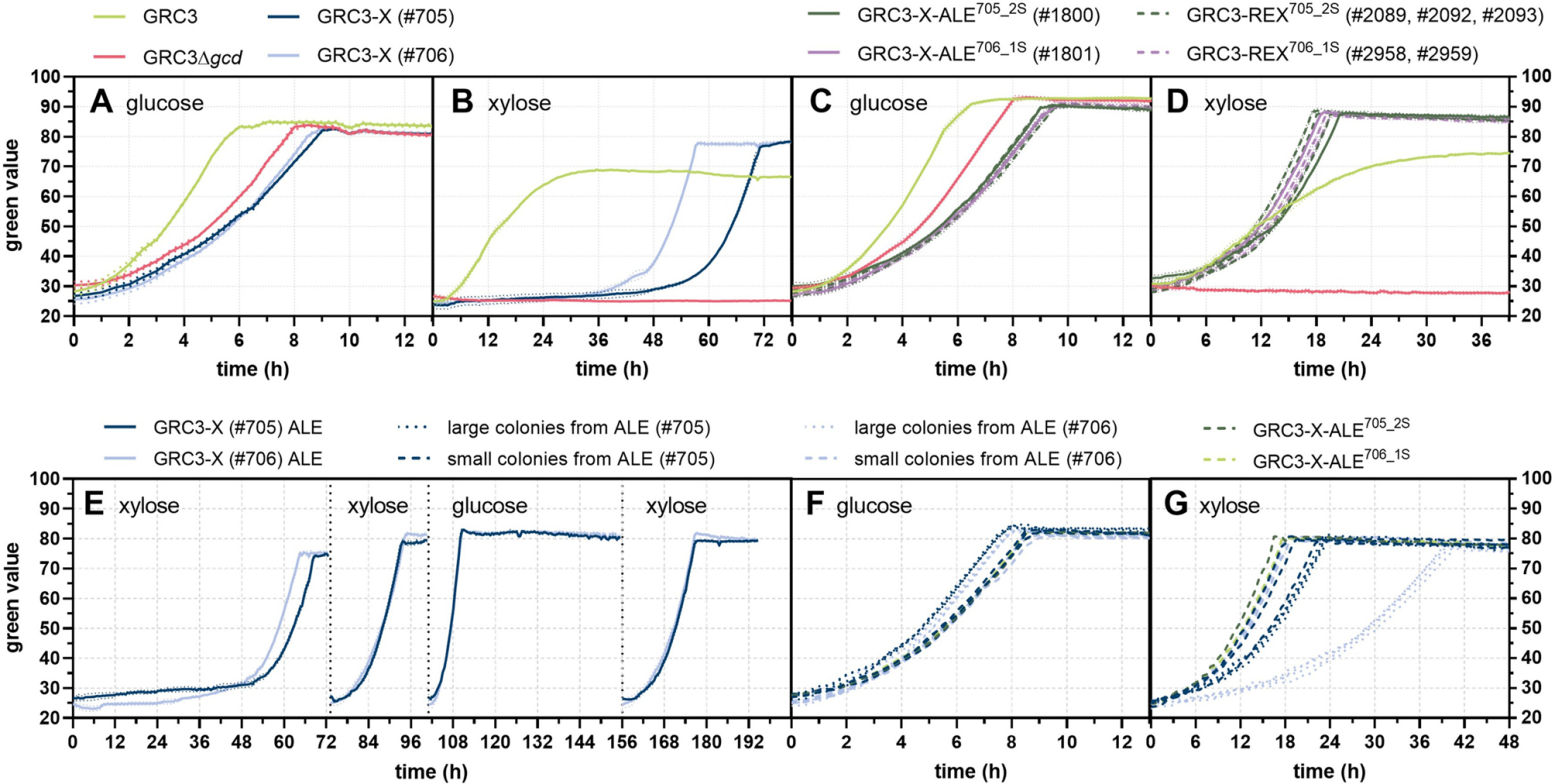
Evaluation of the xylose isomerase pathway in (reverse-)engineered *P. taiwanensis* GRC3 derivatives and sequential-batch ALE campaign for enhanced growth on xylose. Growth of the initially engineered strains on 20 mM glucose (**A**) or 24 mM xylose (**B**) as well as growth of selected ALE mutants and reverse-engineered strains on 20 mM glucose (**C**) or 24 mM xylose (**D**) compared to the reference strains GRC3 and GRC3Δ*gcd*. The experiments were performed in the Growth Profiler 960. The lines represent means of replicates (*n* = 4). The dotted lines indicate the standard deviation. Panel **E** shows sequential-batch ALE cultivations for two independent clones of strain GRC3-X (#705 and #706). Both clones were cultivated in replicates (*n* = 4) using the Growth Profiler 960. The initial replicates were inoculated to an OD_600_ of 0.1 from one glucose-grown pre-culture. During the ALE, 5 µL of each well was transferred to the next well to inoculate 195 µL of fresh medium. For the first, second and fourth batch MSM with 24 mM xylose was used. For the third batch MSM with 20 mM glucose was used. Transfers between the four batches are indicated by black dotted vertical lines. Growth of the evolved clones was evaluated in MSM with 20 mM glucose (**F**) or 24 mM xylose (**G**). The lines represent means of replicates (*n* = 4). The dotted lines indicate the standard deviation

As expected, the deletion of *gcd* resulted in a significantly reduced growth rate on glucose (0.39 ± 0.01 vs. 0.59 ± 0.05 h^−1^ of GRC3, *p* < 0.01) (Fig. 3A) and entirely impaired growth on xylose (Fig. 3B). An overview of all estimated growth rates can be found in the Supplementary information (Table S1). The implementation of the xylose isomerase pathway (GRC3-X) slightly delayed growth on glucose (Fig. 3A), possibly due to the constitutive expression of the genetic xylose isomerase pathway cassette resulting in a metabolic burden. However, the constitutive expression of the transaldolase and transketolase could also interfere with the native metabolism, which might result in unfavorable flux distributions on glucose. The implementation of *xylEAB-talB-tktA* also restored growth on xylose (Fig. 3B). In contrast, identical strains lacking *talB-tktA* (designated GRC3-X-short) did not grow on xylose (Figures S1B, S2), underlining the importance of their expression. However, the two independent GRC3-X clones (#705 and #706) showed extended lag phases (Fig. 3B) with significantly different growth rates (*p* < 0.001) (Table S1). This was somewhat unexpected because the identical construct—although in a different genomic context—enabled sufficient growth in *gcd*-deficient *P. putida* KT2440 [18]. The different lag phases and growth rates for the two individual clones of GRC3-X are an indication of the emergence of mutations. For this reason, both clones were subject to a sequential-batch ALE (Fig. 3E) to enhance growth on xylose. The growth performance on glucose and xylose of single colonies derived from the ALE campaign is shown in Fig. 3F and G, respectively. The best-performing colonies, GRC3-X-ALE^705_2S^ and GRC3-X-ALE^706_1S^, resulting from two independent ALE lineages were selected for whole-genome sequencing (WGS). Strikingly, there was only one conspicuous mutation in each clone, and both were located within *xylB* (Table S2). In GRC3-X-ALE^705_2S^ and GRC3-X-ALE^706_1S^ single nucleotide variations (SNVs) appeared that caused an amino acid exchange of tryptophan 402 (Trp402) to arginine (XylB^W402R^) and threonine 255 (Thr255) to alanine (XylB^T255A^), respectively. This was unexpected because not only is the wild-type xylulose kinase XylB functional in *E. coli* but it was also successfully transferred to *P. putida* in multiple other studies. That the SNVs caused loss-of-function mutations of XylB was disproven through the deletion of *xylB* in the background of both ALE mutants that completely lost their capability to grow on xylose as a consequence (Figure S3). Therefore, the SNVs were replicated through integration of SNV-adjusted *xylEAB-talB-tktA* constructs in GRC3Δ*gcd*. The resulting reverse-engineered xylose (REX) strains, GRC3-REX^705_2S^ with *xylB*^W402R^ and GRC3-REX^706_1S^ with *xylB*^T255A^, reproduced the enhanced growth of the respective ALE mutants on xylose (Fig. 3D). The growth performance of the evolved and reverse-engineered strains on glucose were very similar (Fig. 3C; Table S1). On xylose, GRC3 showed a Monod-like growth kinetic (Fig. 3D), as reported previously, likely due to bottlenecks within the periplasmic oxidation cascade of xylose to xylonate and the uptake of xylonate [22]. In this context, incomplete carbon source consumption cannot be excluded and would explain the lower biomass signal for GRC3 compared to the evolved and reverse-engineered strains using the xylose isomerase pathway. Estimated growth rates of GRC3 on xylose were determined for the early exponential growth phase and significant differences (*p* < 0.05) were observed, with 0.29 ± 0.05 h^−1^ (Fig. 3B) and 0.18 ± 0.01 h^−1^ (Fig. 3D). This is likely due to the combination of a rather short exponential growth phase at low biomass concentrations followed by Monod-like growth and inaccuracies of the Growth Profiler device. Therefore, estimated growth rates should be interpreted with caution. That said, this was the highest deviation observed for identical strains and conditions between experiments (Table S1). For the *P. taiwanensis* VLB120 wild type, growth rates on xylose were highly dependent on the applied substrate concentration, but were not higher than 0.2 h^−1^ [22], indicating that a growth rate for strain GRC3 of 0.18 h^−1^ is more realistic. The estimated growth rates for the reverse-engineered strains with the xylose isomerase pathway from the same experiment were all slightly yet significantly (*p* < 0.05) higher, ranging from 0.21 to 0.23 h^−1^ (Table S1).

The mechanism behind the *xylB* mutations remains somewhat elusive. However, Thr255 of XylB was previously suggested to be involved in the binding of ATP [33]. Therefore, we used AlphaFold 3 modeling to predict the structure of the XylB homodimer in conjunction with two ATP molecules. Thr255 and Trp402 both showed close vicinity to the ATP binding site (Figure S4) thereby indeed indicating a potential role in the binding of ATP, which likely affects the enzymatic activity. Due to the enhanced growth, the T255A and W402R mutations potentially alleviate a bottleneck at the level of xylulose phosphorylation within the non-oxidative xylose assimilation pathway. However, since the accumulation of sugar phosphates has been reported to be toxic [34], the mutations could also have the opposite effect, reducing XylB activity and thereby decreasing accumulation of xylulose phosphate. The latter explanation is supported by the fact that growth on xylose via the isomerase pathway was only achieved upon evolution of strains that co-expressed the transaldolase (TalB) and transketolase (TktA) from *E. coli*. These enzymes are involved in the conversion of xylulose phosphate within the pentose phosphate pathway thereby likely also alleviating xylulose phosphate accumulation. The accumulation of xylulose phosphate at the expense of ATP or the substrate-independent ATP-hydrolyzing activity of XylB could also interfere with ATP homeostasis [33, 35]. The assumption that the mutations observed in XylB reduce the enzymatic activity is supported by recent findings for evolved *P. putida* KT2440 expressing the xylose isomerase pathway [36]. In the associated study, Woo et al. [36] evolved the respective *P. putida* strain on xylose and mixed sugars (containing glucose, xylose, and arabinose) and observed mutations in XlyB that—although different (i.e., G395W, G395R, G396S, or G396C)—also likely affected ATP binding. Further, the authors demonstrated that the enzyme variants XylB^G395W^ and XylB^G395R^ that appeared under xylose-selective conditions showed significantly reduced xylulose kinase and substrate-independent ATPase activity.

**Fig. 4:**
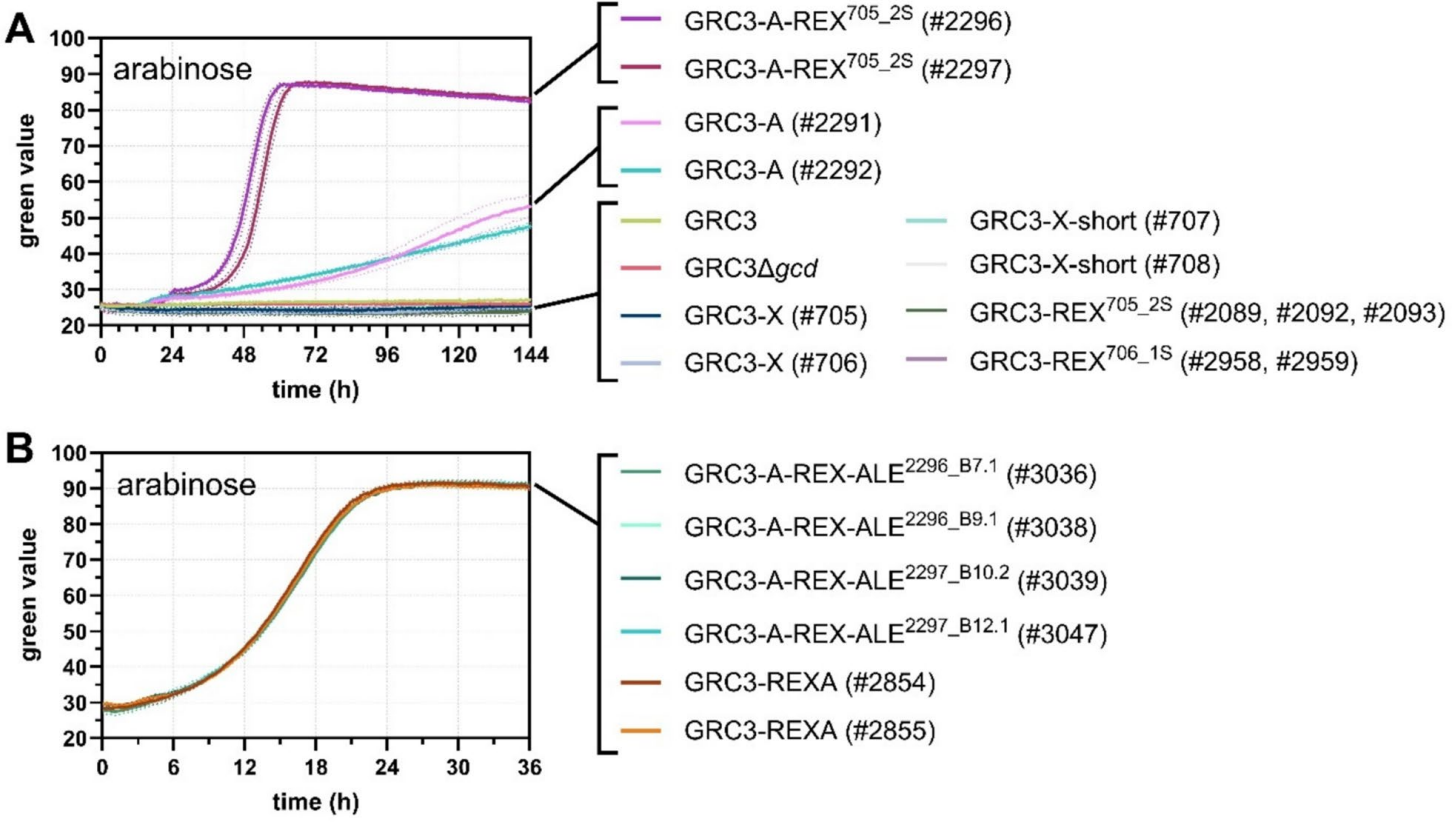
Evaluation of non-oxidative arabinose catabolism in (reverse-)engineered *P. taiwanensis* GRC3 derivatives. Growth of initial strains (**A**), as well as evolved and reverse-engineered strains (**B**), on 24 mM arabinose. The experiment was performed in the Growth Profiler 960. The lines represent means of replicates (*n* = 4). The dotted lines indicate the standard deviation

After sufficient growth on xylose via the xylose isomerase pathway was established, a potential l-arabinose assimilation was investigated. Unlike xylose, arabinose is not metabolized by *P. taiwanensis* VLB120 [37]. Accordingly, strains GRC3 and GRC3Δ*gcd* failed to grow when arabinose was provided as sole carbon source (Fig. 4A). Previously, the implementation of XylA and XylB into *P. putida* was reported to also enable growth on arabinose [12, 17], while this was not the case for the strain engineered by Elmore et al. [18]. None of our strains engineered to metabolize xylose via the isomerase pathway were able to grow on arabinose (Fig. 4A). For this reason, a genetic construct was assembled for the genomic integration of *araECBAD* encoding the non-oxidative arabinose pathway from *E. coli* K-12 MG1655 into the intergenic locus of PVLB_06910/15 (Figure S1C). In the chromosome of *E. coli*, the transporter-encoding *araE* gene is distal to the *araCBAD* gene cluster. For integration into *P. taiwanensis*, the two segments were fused to each other but insulated by bidirectional terminators. The native transcriptional regulation elements of the *araCBAD* cluster from *E. coli* were retained as the *araC-P*_*araBAD*_ expression system was previously shown to be functional and inducible in *Pseudomonas* [38]. The native promoter of *araE*, encoding a low-affinity, high-capacity arabinose/H^+^ symporter, was also retained for AraC-dependent, arabinose-inducible expression [39–41]. The arabinose module was integrated into strains GRC3 and GRC3-REX^705_2S^, resulting in the generation of GRC3-A and GRC3-A-REX^705_2S^, respectively. Two individual clones of GRC3-A (#2291, #2292) showed only very slow growth on 24 mM arabinose with low biomass signals after an extended cultivation time of >6 days (Fig. 4A). In contrast to that, GRC3-A-REX^705_2S^ cultures (#2296, #2297) showed distinctly faster growth (0.15 ± 0.00 and 0.16 ± 0.01 h^−1^), although only after extended lag phases of different durations. The cultivation of GRC3-A-REX^705_2S^ (#2296 and #2297) on arabinose was repeated with a similar trend (Figure S5A), and single colonies of arabinose-grown GRC3-A-REX^705_2S^ (#2296 and #2297) were isolated and re-characterized for their growth performance (Figure S5B). All evolved clones isolated from this one-batch ALE showed faster growth, and most of them reached stationary phase within only 30 h of cultivation. Four of the fastest-growing GRC3-A-REX-ALE clones (namely, 2296_B7.1, 2296_B9.1, 2297_B10.2, and 2297_B12.1) were selected for WGS. All of them harbored nonsense or frameshift mutations in *araE* (Table S2), indicating that expression of arabinose/H^+^ symporter AraE causes a growth impairment under arabinose-induced conditions while the presence of AraE was not disadvantageous for glucose-grown cultures (Figure S6; Table S1). For reverse engineering, a new expression cassette was constructed lacking the *araE* gene and the respective promoter (Figure S1D). Through its introduction into GRC3-REX^705_2S^, the reverse-engineered xylose and arabinose (REXA) strain was obtained. The GRC3-REXA strain (clone #2854 and #2855) showed a growth rate of 0.20 ± 0.00 h^−1^ on arabinose and resembled the growth phenotype of the GRC3-A-REX-ALE clones (#3036, #3038, #3039, and #3047), which all displayed almost identical growth profiles (Fig. 4B). This proves that indeed loss-of-function mutations in *araE* were beneficial, likely by preventing excessive transporter expression. This also shows that there must be an alternative uptake system for arabinose. Desai and Rao [42] reported that the XylE xylose transporter from *E. coli* may also import arabinose and thus, arabinose uptake may be enhanced in the background of the XylE-expressing GRC3-REX^705_2S^ strain. However, the *araC-P*_*araBAD*_ expression system was shown to be inducible in *P. putida* KT2440 even in the absence of the heterologous XylE and AraE transporters, thereby indicating the presence of a native uptake system in *Pseudomonas* [38].

### 4-Coumarate production from lignocellulosic sugars and mock hydrolysates

After heterologous non-oxidative xylose and arabinose utilization was established in the *P. taiwanensis* GRC3-REXA *chassis* strain, the required modifications were integrated into tyrosine-overproducing platform strains to ultimately enable 4-coumarate de novo production from these lignocellulosic pentoses. For this, the *xylEAB*^W402R^*-talB-tktA* and *araCBAD* cassettes were successively integrated into the chromosome of GRC3Δ6-TYR2 and GRC3Δ6-TYR2Δ*ppc*. Subsequently, *gcd* was deleted in the strains harboring only the xylose module and in strains processing both modules. Then, *RpcTAL* was introduced into the Tn7 locus (*attTn7*::*P*_*14f*_*-RpcTAL*) to yield 4-coumarate producers. In contrast to the original 4-coumarate-producing reference strains (Δ5) described above, the tyrosine platform REX(A) strains additionally harbor a Δ*benABCD* modification (Δ6), which prevents benzoate degradation. In the context of 4-coumarate de novo biosynthesis, this deletion is functionally neutral and thus Δ5 and Δ6 strains are directly comparable.

**Fig. 5:**
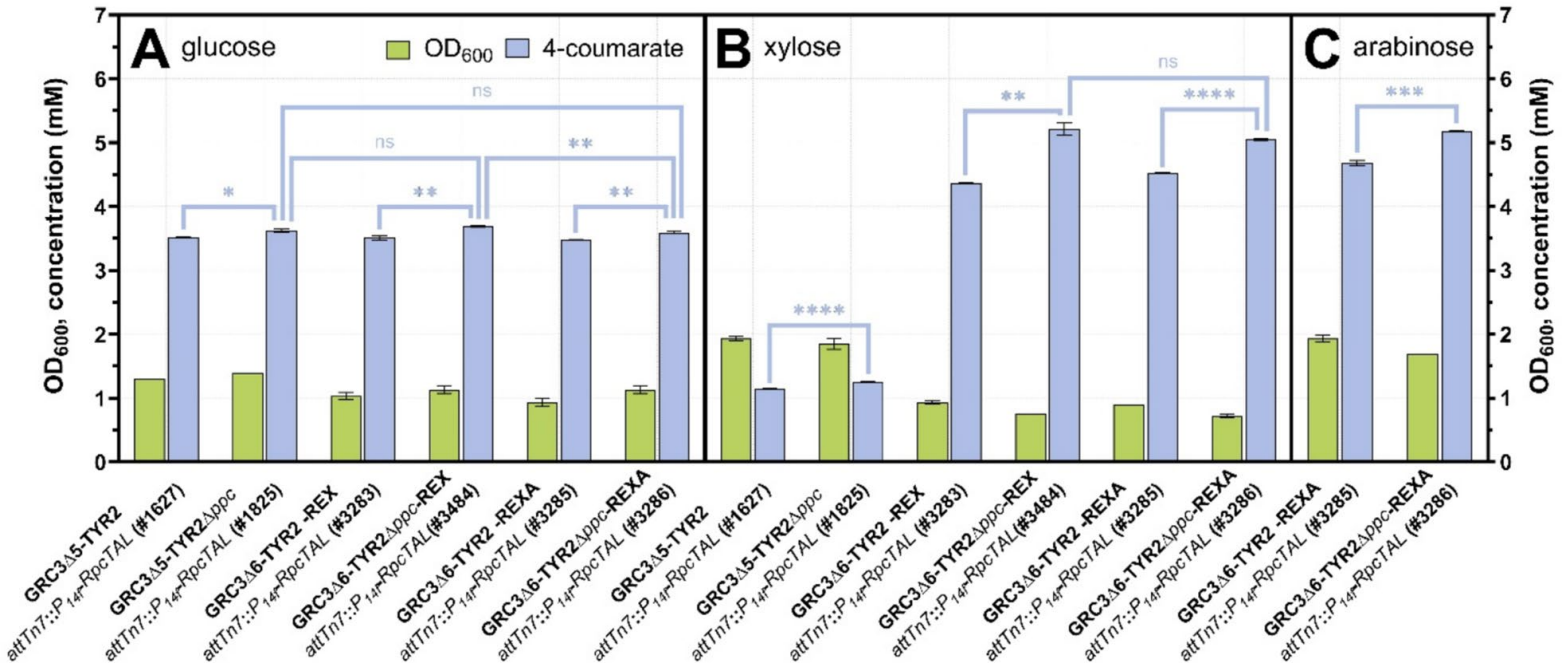
4-Coumarate production from different lignocellulosic sugars. Production was assessed in small-scale 24-well plate cultures in twofold-buffered MSM with 20 mM glucose after 120 h (**A**), 24 mM xylose after 168 h (**B**), and 24 mM arabinose after 192 h (**C**). Pre-cultures were grown with the same carbon source as the main culture in the Growth Profiler 960 using the SIGHT system [43]. Mean values were calculated from a dataset of three biological replicates (*n* = 3). Error bars represent the standard deviation. Statistical significance was evaluated by *t*-tests with *p* < 0.05 (*), *p* < 0.01 (**), *p* < 0.001 (***), *p* < 0.0001 (****) or not significant (ns)

Due to modifications in the central carbon metabolism, an enhanced flux towards tyrosine, and the burden of strong heterologous gene expression, the producer strains’ growth performances are expected to be compromised compared to those of the GRC3-REXA *chassis*. Therefore, growth of the different producers was evaluated on glucose, xylose, and arabinose as individual sole carbon sources (Figure S7), which helped to choose appropriate cultivation times for end point production screenings on the individual carbon sources (Fig. 5). Compared to the original producers without engineered xylose and arabinose metabolism, growth of the REX and REXA strains on glucose was slower likely due to the deletion of *gcd*, which prevents periplasmic glucose oxidation (Figure S7A). Importantly however, 4-coumarate titers remained unaffected by the engineering for non-oxidative xylose and arabinose utilization with 3.5 mM for the strains lacking, and 3.6 mM for the strain harboring the Δ*ppc* modification (Fig. 5A; Table 2). On xylose, the REX and REXA producers without Δ*ppc* showed enhanced growth characteristics compared to the respective reference strain still possessing the Weimberg pathway, while those with Δ*ppc* showed slightly delayed growth (Figure S7B). The major benefit of the REX and REXA strains was the significantly enhanced product titers (Fig. 5B; Table 2) that showed a 3.7- to 4-fold increase compared to the respective reference strain with native xylose metabolism, likely due to enhanced provision of E4P for the shikimate pathway. Notably, the producers harboring the native Weimberg pathway showed incomplete carbon source consumption (xylose/xylonate) (Table 2). Even when this was taken into account in the yield calculation, GRC3Δ5-TYR2-*attTn7*::*P*_*14f*_*-RpcTAL* (#1627) and GRC3Δ5-TYR2Δ*ppc*-*attTn7*::*P*_*14f*_*-RpcTAL* (#1825) only supported product yields of 9.5 ± 0.1 and 10.8 ± 0.0% (Cmol/Cmol) on xylose, respectively. The respective REXA strains without and with Δ*ppc* enabled yields of 34.2 ± 0.1 and 38.2 ± 0.1% (Cmol/Cmol) (Table 2). This clearly demonstrates the superiority of the xylose isomerase pathway over the Weimberg pathway in the context of aromatics production. Growth of the REXA producers on arabinose (Figure S7C) was slower than on xylose despite a similar assimilation pathway and entry into the pentose phosphate pathway (xylulose 5-phosphate). Additionally, minor residual arabinose concentrations were detected after an extended cultivation time of 192 h (Table 2). Nevertheless, very similar 4-coumarate titers and yields were achieved on arabinose (Fig. 5C) in comparison to the individual strains on xylose, showcasing that both pentoses were successfully integrated for efficient 4-coumarate production.

**Table 2:** Key performance indicators for the production of 4-coumarate from glucose, xylose, arabinose and mixtures of these sugars

Strain	Carbon source	4-Coumarate titer (mM)	Residual carbon source (mM)	Yield (%)^a^	Fig
GRC3Δ5-TYR2 *attTn7*::*P*_*14f*_*-RpcTAL* (#1627)	20 mM glucose	3.52 ± 0.01 (120 h)	Not detected	25.5 ± 0.0 (120 h)	5A
GRC3Δ5-TYR2Δ*ppc attTn7*::*P*_*14f*_*-RpcTAL* (#1825)	20 mM glucose	3.63 ± 0.03 (120 h)	Not detected	26.2 ± 0.2 (120 h)	5A
GRC3Δ6-TYR2-REX *attTn7*::*P*_*14f*_*-RpcTAL* (#3283)	20 mM glucose	3.51 ± 0.03 (120 h)	Not detected	25.4 ± 0.2 (120 h)	5A
GRC3Δ6-TYR2Δ*ppc*-REX *attTn7*::*P*_*14f*_*-RpcTAL* (#3484)	20 mM glucose	3.69 ± 0.01 (120 h)	Not detected	26.7 ± 0.1 (120 h)	5A
GRC3Δ6-TYR2-REXA *attTn7*::*P*_*14f*_*-RpcTAL* (#3285)	20 mM glucose	3.48 ± 0.01 (120 h)	Not detected	25.1 ± 0.0 (120 h)	5A
GRC3Δ6-TYR2Δ*ppc*-REXA *attTn7*::*P*_*14f*_*-RpcTAL* (#3286)	20 mM glucose	3.59 ± 0.02 (120 h)	Not detected	26.0 ± 0.1 (120 h)	5A
GRC3Δ5-TYR2 *attTn7*::*P*_*14f*_*-RpcTAL* (#1627)	24 mM xylose	1.15 ± 0.00 (168 h)	2.14 ± 0.15^b^ (168 h)	9.5 ± 0.1 (168 h)	5B
GRC3Δ5-TYR2Δ*ppc attTn7*::*P*_*14f*_*-RpcTAL* (#1825)	24 mM xylose	1.26 ± 0.00 (168 h)	2.93 ± 0.03^b^ (168 h)	10.8 ± 0.0 (168 h)	5B
GRC3Δ6-TYR2-REX *attTn7*::*P*_*14f*_*-RpcTAL* (#3283)	24 mM xylose	4.37 ± 0.01 (168 h)	Not detected	33.0 ± 0.1 (168 h)	5B
GRC3Δ6-TYR2Δ*ppc*-REX *attTn7*::*P*_*14f*_*-RpcTAL* (#3484)	24 mM xylose	5.21 ± 0.09 (168 h)	Not detected	39.4 ± 0.7 (168 h)	5B
GRC3Δ6-TYR2-REXA *attTn7*::*P*_*14f*_*-RpcTAL* (#3285)	24 mM xylose	4.52 ± 0.01 (168 h)	Not detected	34.2 ± 0.1 (168 h)	5B
GRC3Δ6-TYR2Δ*ppc*-REXA *attTn7*::*P*_*14f*_*-RpcTAL* (#3286)	24 mM xylose	5.05 ± 0.01 (168 h)	Not detected	38.2 ± 0.1 (168 h)	5B
GRC3Δ6-TYR2-REXA *attTn7*::*P*_*14f*_*-RpcTAL* (#3285)	24 mM arabinose	4.68 ± 0.04 (192 h)	1.31 ± 0.39 (192 h)	37.6 ± 0.4 (192 h)	5C
GRC3Δ6-TYR2Δ*ppc*-REXA *attTn7*::*P*_*14f*_*-RpcTAL* (#3286)	24 mM arabinose	5.18 ± 0.02 (192 h)	0.20 ± 0.02 (192 h)	39.7 ± 0.1 (192 h)	5C
GRC3Δ5-TYR2Δ*ppc attTn7*::*P*_*14f*_*-RpcTAL* (#1825)	13 mM glucose 7 mM xylose 1 mM arabinose	2.79 ± 0.04 (168 h)	0.11 ± 0.03 arabinose (168 h)	21.1 ± 0.3 (168 h)	6A
GRC3Δ6-TYR2Δ*ppc*-REXA *attTn7*::*P*_*14f*_*-RpcTAL* (#3286)	13 mM glucose 7 mM xylose 1 mM arabinose	3.45 ± 0.01 (168 h)	0.05 ± 0.01 arabinose (168 h)	26.4 ± 0.6 (168 h)	6A
GRC3Δ5-TYR2Δ*ppc attTn7*::*P*_*14f*_*-RpcTAL* (#1825)	10 mM glucose 9 mM xylose 3 mM arabinose	2.34 ± 0.00 (168 h)	0.31 ± 0.03 xylose^b^ 0.80 ± 0.02 arabinose (168 h)	18.5 ± 0.0 (168 h)	6B
GRC3Δ6-TYR2Δ*ppc*-REXA *attTn7*::*P*_*14f*_*-RpcTAL* (#3286)	10 mM glucose 9 mM xylose 3 mM arabinose	4.21 ± 0.01 (168 h)	Not detected	32.1 ± 0.2 (168 h)	6B

^a^Yield is given as carbon-molar percentage (% (Cmol/Cmol))

^b^Combined concentration of xylose and xylonate as no analytic discrimination was possible

**Fig. 6:**
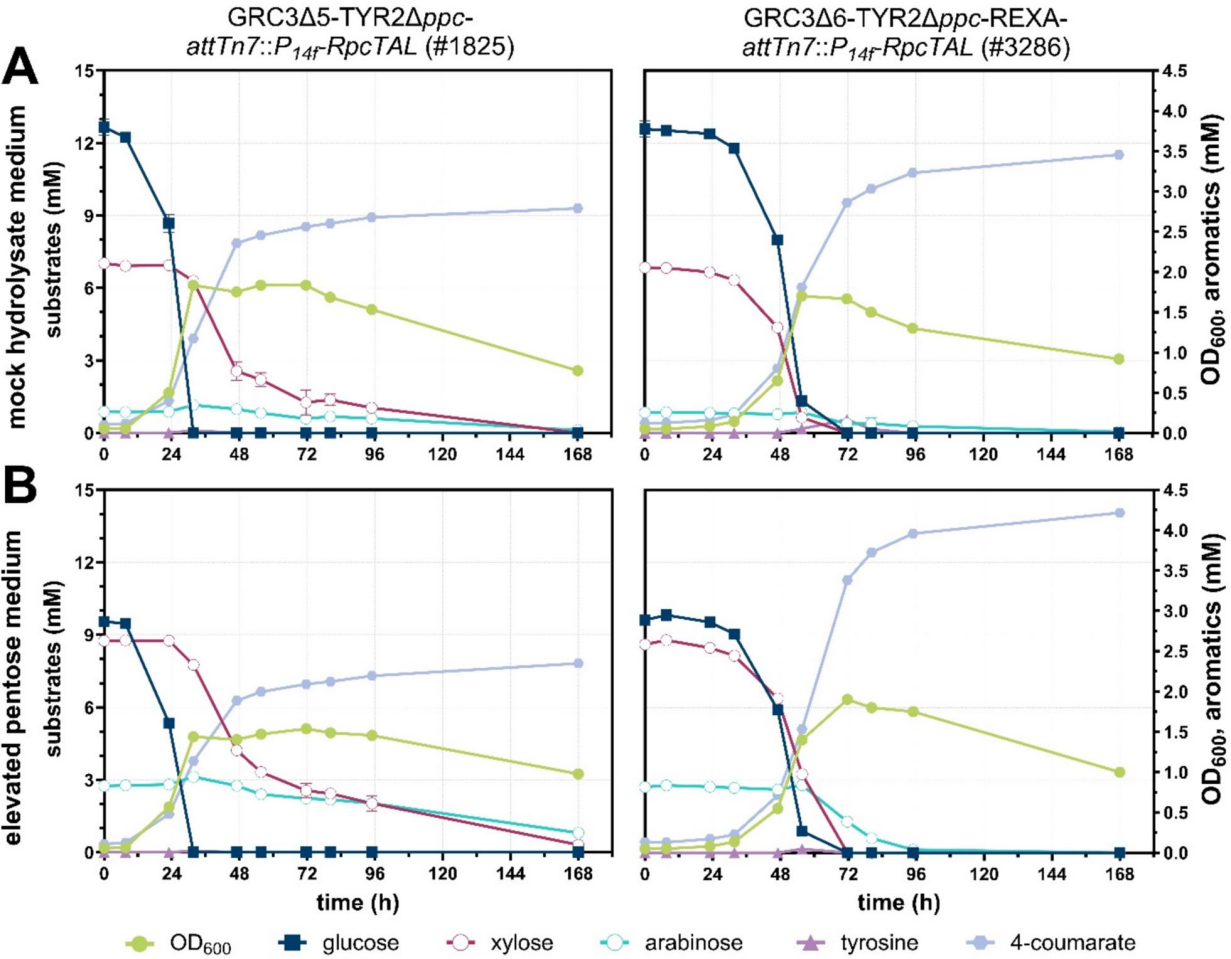
Shake flask cultivations of GRC3Δ5-TYR2Δ*ppc*-*attTn7*::*P*_*14f*_*-RpcTAL* (#1825) and GRC3Δ6-TYR2Δ*ppc*-REXA-*attTn7*::*P*_*14f*_*-RpcTAL* (#3286) for the production of 4-coumarate in mineral salt medium (twofold buffered) containing either 13 mM glucose, 7 mM xylose, and 1 mM arabinose (**A**) or 10 mM, 9 mM xylose, and 3 mM arabinose (**B**). GRC3Δ5-TYR2Δ*ppc*-*attTn7*::P_14f_-*RpcTAL* oxidizes glucose and xylose to gluconate and xylonate, respectively. The plotted glucose and xylose concentrations potentially include the respective sugar acid as they could not be discriminated from the respective sugar due to analytic limitations. Mean values were calculated from a dataset of three biological replicates (*n* = 3). Error bars represent the standard deviation

Subsequently, we characterized growth and production of selected strains on mixtures of glucose, xylose, and arabinose. We applied these sugars in a ratio roughly representing that of hydrolyzed corn stover [18, 44, 45], using mock hydrolysate medium with 13 mM glucose, 7 mM xylose, and 1 mM arabinose (Fig. 6A). Additionally, a medium with an elevated pentose availability was tested, with a mixture of 10 mM glucose, 9 mM xylose, and 3 mM arabinose (Fig. 6B). Strain GRC3Δ6-TYR2Δ*ppc*-REXA-*attTn7*::*P*_*14f*_*-RpcTAL* (#3286) was selected for further characterization because this strain metabolizes all three sugars and the Δ*ppc* modification was found to significantly enhance production especially on the pentoses. GRC3Δ5-TYR2Δ*ppc*-*attTn7*::*P*_*14f*_*-RpcTAL* (#1825) served as reference strain. Performance indicators for associated cultivations are shown in Table 2.

The reference strain consumed glucose significantly faster than the REXA producer, within 32 instead of 72 h, and also showed faster growth. GRC3Δ5-TYR2Δ*ppc*-*attTn7*::*P*_*14f*_*-RpcTAL* (#1825) started to consume xylose only upon the depletion of glucose, and xylose consumption rates drastically decreased after 47 h. For this strain, complete xylose consumption was only achieved in the mock hydrolysate medium (Fig. 6A) after an extended cultivation time (168 h), while a low concentration of 0.31 ± 0.03 mM xylose (potentially including xylonate) still remained in the medium with the elevated pentose concentrations (Fig. 6B). In both media, arabinose concentrations remained relatively stable in the first 47 h of cultivation, but slowly decreased thereafter, to only 12.8 ± 2.9 and 29.2 ± 0.8% (after 168 h) of the initial concentration of the mock hydrolysate and elevated pentose medium, respectively. This was unexpected, since this strain does not possess a native arabinose assimilation route as shown before, and the phenomenon could be associated to the slow conversion to a dead-end product. Considering the consumption of glucose, xylose, and also arabinose, GRC3Δ5-TYR2Δ*ppc*-*attTn7*::*P*_*14f*_*-RpcTAL* (#1825) achieved a 4-coumarate yield of 21.1 ± 0.3% (Cmol/Cmol) and a titer of 2.79 ± 0.04 mM in the mock hydrolysate medium (Fig. 6A). In the medium with elevated pentose concentrations, yield and titer were slightly lower with 18.5 ± 0.0% (Cmol/Cmol) and 2.34 ± 0.00 mM, respectively (Fig. 6B).

Although, the initial growth and glucose consumption of GRC3Δ6-TYR2Δ*ppc*-REXA-*attTn7*::*P*_*14f*_*-RpcTAL* (#3286) was slower, this strain co-metabolized glucose and xylose simultaneously due to constitutive expression of the xylose isomerase pathway, and xylose was fully consumed within only 72 h. Arabinose utilization appeared only after 56 h, when glucose was nearly depleted, likely due to the native regulation for the non-oxidative arabinose pathway. By the end of the cultivation, arabinose was depleted in the elevated pentose medium (Fig. 6B), while a very low residual arabinose concentration (0.05 ± 0.01 mM) was observed for the mock hydrolysate medium (Fig. 6A). With regard to production, GRC3Δ6-TYR2Δ*ppc*-REXA-*attTn7*::*P*_*14f*_*-RpcTAL* (#3286) achieved yields of 26.4 ± 0.6 and 32.1 ± 0.2% (Cmol/Cmol) and titers of 3.45 ± 0.01 and 4.21 ± 0.01 mM 4-coumarate in the mock hydrolysate (Fig. 6A) and elevated pentose medium (Fig. 6B), respectively. The achieved yields translate into an improvement of 25.1 ± 2.8% in the mock hydrolysate medium and 73.9 ± 1.3% under elevated pentose concentrations, when comparing the REXA producer to the reference strain. This again demonstrates the benefit of non-oxidative utilization of pentoses for the production of aromatics from a mixture of lignocellulosic sugars.

## Conclusion

In the present study, we first significantly enhanced 4-coumarate production compared to a previously published *P. taiwanensis* strain through the application of a tyrosine-specific ammonia-lyase from *Rivularia* sp. PCC7116 (*Rpc*TAL) and the deletion of *ppc* to enhance the phosphoenolpyruvate availability. Thereby, we could achieve yields of 28.2 and 37.0% (Cmol/Cmol) from glucose and glycerol, respectively.

Subsequently, we extended our efforts to enable sufficient 4-coumarate production from lignocellulosic pentoses to allow for an optimized valorization of agro-industry side streams. Although non-oxidative xylose and arabinose pathways were established in other *Pseudomonas* species before, we observed that the important mutations were all localized in the heterologous assimilation modules, even for the xylose utilization cassette that was previously successfully applied in *P. putida*. This demonstrates that the design of heterologous modules is not always trivial and that established cassettes are not always easily transferable between species, even of the same genus. Metabolic burden and bottlenecks need careful consideration and potentially the adjustment of expression cassettes.

Reverse-engineered and optimized assimilation modules were subsequently transferred to aromatics-producing strains for the synthesis of 4-coumarate from the lignocellulosic sugars glucose, xylose, and arabinose. The final producer, GRC3Δ6-TYR2Δ*ppc*-REXA-*attTn7*::*P*_*14f*_*-RpcTAL*, enabled 4-coumarate production from xylose with a yield of 38.2% (Cmol/Cmol), which was a significant improvement compared to the native Weimberg pathway for oxidative xylose utilization that only supported a yield of 10.8% (Cmol/Cmol). On arabinose, GRC3Δ6-TYR2Δ*ppc*-REXA-*attTn7*::*P*_*14f*_*-RpcTAL* showed a similar yield as on xylose with 39.7% (Cmol/Cmol), thereby successfully integrating arabinose as a carbon source for efficient aromatics production. In media containing different mixtures of glucose, xylose, and arabinose, the REXA strain also outperformed its reference regarding titer and yield. However, for this strain, yields were lower on sugar mixtures compared to the application of xylose or arabinose as sole carbon source. This indicates that there is an enhanced provision of precursors for the shikimate pathway when these pentoses are provided to strain GRC3Δ6-TYR2Δ*ppc*-REXA-*attTn7*::*P*_*14f*_*-RpcTAL* via the pentose phosphate pathway, which is hardly active on glucose alone [46].

## Experimental procedures

### Media and culture conditions

Routinely, *Escherichia coli* and *Pseudomonas taiwanensis* were cultured at 37 and 30 °C, respectively, in lysogeny broth (LB) medium comprising 10 g L^−1^ tryptone, 5 g L^−1^ yeast extract, and 5 g L^−1^ sodium chloride. Solid LB medium additionally contained 15 g L^−1^ agar. Sucrose counterselection was performed on plates containing 5 g L^−1^ yeast extract (Merck), 10 g L^−1^ *N*-*Z*-Amine A (casein enzymatic hydrolysate, Sigma-Aldrich), 18 g L^−1^ agar–agar (Carl Roth), and 250 g L^−1^ sucrose (Sigma-Aldrich). Growth experiments were performed using mineral salt medium (MSM) [47] with an adjusted standard buffer concentration (22.3 mM K_2_HPO_4_ and 13.6 mM NaH_2_PO_4_). In production experiments, the buffer concentration was increased twofold. d(+)-Glucose monohydrate (Carl Roth), glycerol (Chemsolute, Th. Geyer), d(+)-xylose (Sigma-Aldrich), and l(+)-arabinose (Carl Roth) served as carbon sources, as indicated for the individual experiments. Antibiotics were added to the medium to ensure selective conditions as required. Ampicillin sodium salt (Carl Roth) was used at a concentration of 100 mg L^−1^ for *E. coli* DH5α λpir pTNS1. Kanamycin sulfate (Carl Roth) was applied at a concentration of 50 mg L^−1^, and gentamicin (Carl Roth) at 10 mg L^−1^. However, in LB agar, the concentration of gentamicin was increased to 25 mg L^−1^ for *P taiwanensis*. After conjugational mating, 25 mg L^−1^ Irgasan (Sigma-Aldrich) was added to the LB agar plate to select for *P. taiwanensis*. Pre-cultures for growth experiments were grown in MSM containing 20 mM glucose, if not indicated otherwise. Pre-cultures for production experiments were grown in the same medium as the main cultures, but kanamycin was only supplemented to the pre-cultures. Main cultures of production experiments were inoculated from the respective pre-culture to an initial OD_600_ of 0.05, while growth experiments in the Growth Profiler 960 were inoculated to an OD_600_ of 0.1, if not specifically stated differently. Shake flask cultivations were performed in Erlenmeyer flasks with a filling volume of 10% (v/v), in a horizontal rotary shaker with a throw of 50 mm at 200 rpm. Small-scale cultivations and pre-cultures for Growth Profiler 960 experiments were grown in 1.5 mL medium in System Duetz 24-well microtiter plates (CR1424a, EnzyScreen), sealed with sandwich covers (CR1224b, EnzyScreen), that were shaken with 300 rpm and a throw of 50 mm. The pre-cultures for the initial production and growth experiments associated to Fig. 5 and Figure S7 were grown in the Growth Profiler using the SIGHT system [43] using 5-mL vials with a filling volume of 1 mL. For Growth Profiler experiments, greyish-white 96-half-deep-well microtiter plates with flat transparent bottoms (CR1496dg, EnzyScreen) were filled with 200 μL of the cell suspensions, sealed with sandwich covers (CR1296b, EnzyScreen), and shaken at 225 rpm.

### Plasmid cloning and strain engineering

Strains and plasmids used and generated in this study are listed in Table 3 and Table S3, respectively. Plasmids were constructed by Gibson cloning using the NEBuilder HiFi DNA Assembly Master Mix (New England Biolabs), except for plasmid pEMG-*gcd*, that was generated through ligation cloning using the T4 ligase (Thermo Fisher Scientific). PCR amplification of DNA fragments was routinely achieved using the Q5 High-Fidelity 2X Master Mix (New England Biolabs). In rare cases, the Phusion High-Fidelity DNA Polymerase (New England Biolabs) or the Platinum SuperFi II Green PCR Master Mix (Thermo Fisher Scientific) was used to amplify DNA for cloning. Primers used for cloning were ordered as unmodified oligonucleotides from Eurofins Genomics (Table S4). Restriction enzymes were purchased from Thermo Fisher Scientific or New England Biolabs. Additional details on the cloning procedures of specific plasmids can be found in Table S5. The correct assembly of plasmids was confirmed by Sanger sequencing performed by Eurofins Genomics. The tyrosine ammonia-lyase-encoding gene from *Rivularia* sp. PCC7116 (GenBank: CP003549.1; locus tag: Riv7116_3074) was codon-optimized for *P. taiwanensis* VLB120 using OPTIMIZER online tool [48] according to the workflow described in Wynands et al. [4] and ordered as gBlock Gene Fragment (Integrated DNA Technologies). The codon-optimized gene sequence denoted *RpcTAL* can be found in the Supplementary information.

**Table 3:** Bacterial strains used in this study

Strain	Relevant characteristics	Reference
*Escherichia coli*		
DH5α	F^−^ Φ80 *lac*ZΔM15 Δ(*lac*ZYA-*arg*F)*U169 recA1 endA1 hsdR17*(r_k_^−^, m_k_^+^) *phoA supE44 thi-1 gyrA96 relA1* λ^−^; *host for oriV(colE1) plasmids*	Thermo Fischer Scientific
NEB 5-alpha	*fhuA2Δ(argF-lacZ)U169 phoA glnV44 Φ80Δ(lacZ)M15 gyrA96 recA1 relA1 endA1 thi-1 hsdR17; host for oriV(colE1) plasmids*	New England Biolabs
EPI400	F^−^ *mcrA* ∆(*mrr-hsdRMS-mcrBC*) φ80d*lacZ*∆M15 ∆*lacX74 recA1 endA1 araD139* ∆(*ara*, *leu*)*7697 galU galK λ*^−^ *rpsL nupG tonA* ∆*pcnB* (*DHFR*)	Lucigen
DH5α λpir	λpir lysogen of *E. coli* DH5α; host for *oriV(R6K)* plasmids	Martínez-García and de Lorenzo [49]
PIR2	*F*^*−*^ ∆*lac169 rpoS(Am) robA1 creC510 hsdR514 endA recA1 uidA(*∆*MluI)*::*pir*; host for *oriV(R6K)* plasmids	Thermo Fischer Scientific
EC100D *pir*^+^	F^−^ *mcrA* ∆*(mrr-hsdRMS-mcrBC)* Φ80d*lacZ*∆M15 ∆*lacX74 recA1 endA1 araD139* ∆*(ara, leu)7697 galU galK λ*^−^* rpsL nupG pir*^+^*(DHFR)*; host for *oriV(R6K)* plasmids	Lucigen
HB101 pRK2013	F^−^*Δ(gpt-proA)62leuB6glnV44 ara-14 galK2 lacY1 Δ(mcrC-mrr) rpsL20* (Str^R^) *xyl-5 mtl-1 recA13 thi-1* with plasmid pRK2013	Ditta et al. [52]
DH5α λpir pTNS1	DH5α λpir with plasmid pTNS1	Choi et al. [53]
DH5α pSW-2	DH5α with plasmid pSW-2	Martínez-García and de Lorenzo [49]
*Pseudomonas taiwanensis*		
GRC3	Genome-reduced *chassis* strain of VLB120 with ΔpSTY, Δprophage1/2::*ttgVWGHI*, Δprophage3, Δprophage4, Δflag1, Δflag2, Δlap1, Δlap2, Δlap3	Wynands et al. [6] MiKat#5
GRC3Δ*gcd*	GRC3 with Δ*gcd*	This study MiKat #684
GRC3-X	GRC3Δ*gcd* with PVLB_22010/15::*xylEAB-talB-tktA*	This study MiKat #705 MiKat #706
GRC3-X-short	GRC3Δ*gcd* with PVLB_22010/15::*xylEAB*	This study MiKat# 707 MiKat# 708
GRC3-X-ALE^705_2S^	ALE mutant 705_2S derived from strain GRC3-X (#705); selected due to enhanced growth phenotype on xylose	This study MiKat #1800
GRC3-X-ALE^706_1S^	ALE mutant 706_1S derived from strain GRC3-X (#706); selected due to enhanced growth phenotype on xylose	This study MiKat #1801
GRC3-X-ALE^705_2S^Δ*xylB*	GRC3-X-ALE^705_2S^ with Δ*xylB*	This study MiKat #2094
GRC3-X-ALE^706_1S^Δ*xylB*	GRC3-X-ALE^706_1S^ with Δ*xylB*	This study MiKat #2095
GRC3-REX^705_2S^	GRC3-X with reverse-engineered *xylB*^W402R^	This study MiKat #2089 MiKat #2092 MiKat #2093
GRC3-REX^706_1S^	GRC3-X with reverse-engineered *xylB*^T255A^	This study MiKat #2958 MiKat #2959
GRC3-A	GRC3 with PVLB_06910/15::*araECBAD*	This study MiKat #2291 MiKat #2292
GRC3-A-REX	GRC3-REX^705_2S^ with PVLB_06910/15::*araECBAD*	This study MiKat #2296 MiKat #2297
GRC3-A-REX-ALE^2296_B7.1^	ALE mutant 2296_B7.1 derived from strain GRC3-A-REX (#2296); selected due to enhanced growth phenotype on arabinose	This study MiKat #3036
GRC3-A-REX-ALE^2296_B9.1^	ALE mutant 2296_B7.1 derived from strain GRC3-A-REX (#2296); selected due to enhanced growth phenotype on arabinose	This study MiKat #3038
GRC3-A-REX-ALE^2297_B10.2^	ALE mutant 2297_B10.2 derived from strain GRC3-A-REX (#2297); selected due to enhanced growth phenotype on arabinose	This study MiKat #3039
GRC3-A-REX-ALE^2297_B12.1^	ALE mutant 2297_B12.1 derived from strain GRC3-A-REX (#2297); selected due to enhanced growth phenotype on arabinose	This study MiKat #3047
GRC3-REXA	GRC3-REX^705_2S^ with PVLB_06910/15::*araCBAD*	This study MiKat #2854 MiKat #2855
GRC3Δ5-TYR2	GRC3, Δ*pobA*, Δ*hpd*, Δ*quiC*, Δ*quiC1*, Δ*quiC2*, *trpE*^P290S^, *aroF-1*^P148L^, Δ*pykA*	Wynands et al. [9] MiKat #58
GRC3Δ5-TYR2Δ*ppc*	GRC3Δ5-TYR2 with Δ*ppc*	This study MiKat #59
GRC3Δ5-TYR3	GRC3Δ5-TYR2 with *pheA*^P144S^	Wynands et al. [9] MiKat #60
GRC3Δ5-TYR3Δ*ppc*	GRC3Δ5-TYR3 with Δ*ppc*	This study MiKat #61
GRC3Δ6-TYR1	GRC3, Δ*pobA*, Δ*hpd*, Δ*quiC*, Δ*quiC1*, Δ*quiC2*, Δ*benABCD*, *trpE*^P290S^, *aroF-1*^P148L^, Δ*pykA*	Kofler et al. [54] MiKat #836
GRC3Δ6-TYR2-REX	GRC3Δ6-TYR1 with *pheA*^T310I^, Δ*gcd*, PVLB_22010/15:: *xylEAB*^W402R^*-talB-tktA*	This study MiKat #3151
GRC3Δ6-TYR2Δ*ppc*-REX	GRC3Δ6-TYR2-REX with Δ*ppc*	This study MiKat #3154
GRC3Δ6-TYR2-REXA	GRC3Δ6-TYR2-REX with PVLB_06910/15::*araCBAD*	This study MiKat #3157
GRC3Δ6-TYR2Δ*ppc*-REXA	GRC3Δ6-TYR2-REXA with Δ*ppc*	This study MiKat #3159
GRC3Δ5-TYR2-*attTn7*::*P*_*14f*_-*RtPAL*	GRC3Δ5-TYR2 with *attTn7*::*Kan_FRT*-*P*_*14f*_-*RtPAL*	Wynands et al. [9] MiKat #958
GRC3Δ5-TYR2Δ*ppc*-*attTn7*::*P*_*14f*_-*RtPAL*	GRC3Δ5-TYR2Δ*ppc* with *attTn7*::*Kan_FRT*-*P*_*14f*_-*RtPAL*	This study MiKat #1823
GRC3Δ5-TYR3-*attTn7*::*P*_*14f*_-*RtPAL*	GRC3Δ5-TYR3 with *attTn7*::*Kan_FRT*-*P*_*14f*_-*RtPAL*	Wynands et al. [9] MiKat #959
GRC3Δ5-TYR3Δ*ppc*-*attTn7*::*P*_*14f*_-*RtPAL*	GRC3Δ5-TYR3Δ*ppc* with *attTn7*::*Kan_FRT*-*P*_*14f*_-*RtPAL*	This study MiKat #1824
GRC3Δ5-TYR2-*attTn7*::*P*_*14f*_-*RpcTAL*	GRC3Δ5-TYR2 with *attTn7*::*Kan_FRT*-*P*_*14f*_-*RpcTAL*	This study MiKat #1627
GRC3Δ5-TYR2Δ*ppc*-*attTn7*::*P*_*14f*_-*RpcTAL*	GRC3Δ5-TYR2Δ*ppc* with *attTn7*::*Kan_FRT*-*P*_*14f*_-*RpcTAL*	This study MiKat #1825
GRC3Δ6-TYR2-REX-*attTn7*::*P*_*14f*_-*RpcTAL*	GRC3Δ6-TYR2-REX with *attTn7*::*Kan_FRT*-*P*_*14f*_-*RpcTAL*	This study MiKat #3283
GRC3Δ6-TYR2Δ*ppc*-REX-*attTn7*::*P*_*14f*_-*RpcTAL*	GRC3Δ6-TYR2Δ*ppc*-REX with *attTn7*::*P*_*14f*_-*RpcTAL*	This study MiKat #3484
GRC3Δ6-TYR2-REXA-*attTn7*::*P*_*14f*_-*RpcTAL*	GRC3Δ6-TYR2-REXA with *attTn7*::*Kan_FRT*-*P*_*14f*_-*RpcTAL*	This study MiKat #3285
GRC3Δ6-TYR2Δ*ppc*-REXA-*attTn7*::*P*_*14f*_-*RpcTAL*	GRC3Δ6-TYR2Δ*ppc*-REXA with *attTn7*::*Kan_FRT*-*P*_*14f*_-*RpcTAL*	This study MiKat #3286

Gene deletion of *gcd*, *xylB*, and *ppc*, as well as the genomic integration of the arabinose pathway modules *araECBAD* (Figure S1C) and *araCBAD* (Figure S1D) into the intergenic region of PVLB_06910/15 were achieved by homologous recombination, applying the I-SceI counterselection system developed by Martínez-García and de Lorenzo [49], following the streamlined workflow described by Wynands et al. [4]. For this method, pEMG-, pSNW2-, or pBNW2-derived suicide vector were applied (Table S3). pSW-2 served as I-SceI expression plasmid. The integration of the xylose pathway modules *xylEAB-talB-tktA* (Figure S1A) and *xylEAB* (Figure S1B) into the intergenic region of PVLB_22010/15 was also achieved by homologous recombination, using pK19*mobsacB*-derived suicide plasmids (Table S3), applying sucrose counterselection according to the workflow described by Johnson and Beckham [50]. *RtPAL* and *RpcTAL* expression cassettes were site-specifically integrated into the chromosome at the Tn7 attachment site (*attTn7*) using mini-Tn7 transposon plasmids derived from pBG14f*_FRT_Kan* [27] (Table S3), in a procedure as described in Wynands et al. [9]. For all genetic engineering strategies described above, the plasmids were delivered to *P. taiwanensis* from *E. coli* through conjugational patch matings, as described in Wynands et al. [4]. These matings comprised the *E. coli* donor, the *P. taiwanensis* recipient, and the helping strain *E. coli* HB101 pRK2013. For the mini-Tn7 transposon delivery, *E. coli* DH5α λpir pTNS1 was further added to the mating, as this strain provides the required transposase *in trans*. Diagnostic colony PCRs were performed to screen for desired recombination events using One*Taq* QuickLoad 2X Master Mix with Standard Buffer (New England Biolabs). For this, template cell material was pre-lysed in alkaline PEG according to Chomczynski and Rymaszewski [51].

### Whole-genome sequencing

Genomic DNA of selected ALE strains was isolated from overnight LB cultures using the Monarch Genomic DNA Purification Kit (New England Biolabs) and subsequently sequenced on an Illumina MiSeq platform. Sample preparation, sequencing, and data analysis was performed as described in Ackermann et al. [55].

### Protein 3D structure modeling

The three-dimensional structure of XylB from *E. coli* was modeled as a homodimer with two ATP molecules as ligands using AlphaFold 3 [56]. The derived model was visualized using PyMOL.

### Analytical methods

Optical densities of cell suspensions were determined at 600 nm (OD_600_) using an Ultrospec 10 photometer (Biochrom). Online growth curves were acquired using the Growth Profiler 960 and the associated GP960Viewer software (EnzyScreen), which determines green values (intensity of green pixels) from bottom-up pictures of microtiter plates as a proxy for biomass formation in a non-linear correlation to OD_600_. For the calculation of growth rates from Growth Profiler 960 experiments, green values were converted to OD_600_ equivalents using the calibration by Kofler et al. [54]. Culture supernatants were sampled and stored at −20 °C until HPLC analysis, to detect and quantify substrates and aromatics. HPLC analysis was performed using 1260 Infinity II systems equipped with 1260 DAD WR and 1260 RI detectors (Agilent Technologies). Tyrosine, 4-coumarate, and *trans*-cinnamate were detected and quantified using a reversed-phase InfinityLab Poroshell 120 EC-C18 column (3.0 × 150 mm, 2.7 μm, Agilent Technologies, P.N. 693975-302T) equipped with a guard column (Agilent Technologies; P.N.: 823750-911) using the procedure previously published in Wynands et al. [9]. Solutions of l-tyrosine (≥99%, Sigma-Aldrich), 4-coumaric acid (≥98%, Sigma-Aldrich), and *trans*-cinnamic acid (≥99%, Sigma-Aldrich) served as authentic analytical standards. Carbon sources were analyzed using a Metab-AAC (300 × 7.8 mm, ISERA, P.N.: A1BF-A1AA0N) column equipped with a Guard Cartridge Holder (ISERA, P.N.: AA13-000005) and Guard Column (10 × 7.8 mm, ISERA, P.N.: A1BF-A1AG0N) which was eluted with 5 mM H_2_SO_4_ at a flow of 0.6 mL min^−1^ and a temperature of 50 °C for 20 min. d-(+)-Glucose monohydrate (Carl Roth), d-gluconic acid sodium salt (Sigma-Aldrich), d-(+)-xylose (Sigma-Aldrich), d-xylonic acid lithium salt (≥95.0% purity, Sigma-Aldrich), l-(+)-arabinose (Carl Roth), and glycerol (Chemsolute, Th. Geyer) served as authentic standards. Monosaccharides and glycerol were analyzed using the RI detector. Gluconate and xylonate were analyzed using the DA detector, because they co-elute with the respective monosaccharide. Gluconate concentrations were subtracted from that of glucose to obtain corrected glucose concentrations for the data associated to Fig. 2C. For following experiments (data shown in Table 2; Fig. 6), however, glucose and xylose concentrations were determined using the RI detector without subtraction of the respective sugar acid due to analytical limitations of the UV chromatograms.

For the calculation of 4-coumarate yields, the initial and final concentrations as well as the overall carbon source consumption were considered, as determined by HPLC. Growth rates were estimated by applying exponential fits to the OD_600_ data during exponential growth (with *R*^2^ > 0.98). Statistical significance was assessed by *t*-test (two-tailed distribution, heteroscedastic) with the indicated significance levels. *p* values >0.05 were considered as not significant.

## Supplementary Information

Additional file 1.

## Data Availability

Data will be made available from the corresponding author on request.

## References

[1] Huccetogullari D, Luo ZW, Lee SY. Metabolic engineering of microorganisms for production of aromatic compounds. Microb Cell Fact. 2019;18(1):41. 30808357 10.1186/s12934-019-1090-4 PMC6390333

[2] Covestro AG. World’s first pilot plant for bio-based aniline. Press release. 2024. Available from https://www.covestro.com/press/download/3556b541-02bc-455e-a915-22bde46f4922/240213-inauguration-pilotplantbio-basedaniline.pdf.

[3] Jaeger G, Magnus J, Moussa AS. Production of aniline via anthranilate. Patent: US 10173969B2. 2019.

[4] Wynands B, Lenzen C, Otto M, Koch F, Blank LM, Wierckx N. Metabolic engineering of *Pseudomonas taiwanensis* VLB120 with minimal genomic modifications for high-yield phenol production. Metab Eng. 2018;47:121–33. 29548982 10.1016/j.ymben.2018.03.011

[5] Lenzen C, Wynands B, Otto M, Bolzenius J, Mennicken P, Blank LM, et al. High-yield production of 4-hydroxybenzoate from glucose or glycerol by an engineered *Pseudomonas taiwanensis* VLB120. Front Bioeng Biotechnol. 2019;7:130. 31245364 10.3389/fbioe.2019.00130 PMC6581684

[6] Wynands B, Otto M, Runge N, Preckel S, Polen T, Blank LM, et al. Streamlined *Pseudomonas taiwanensis* VLB120 chassis strains with improved bioprocess features. ACS Synth Biol. 2019;8(9):2036–50. 31465206 10.1021/acssynbio.9b00108

[7] Otto M, Wynands B, Lenzen C, Filbig M, Blank LM, Wierckx N. Rational engineering of phenylalanine accumulation in *Pseudomonas taiwanensis* to enable high-yield production of *trans*-cinnamate. Front Bioeng Biotechnol. 2019;7:312. 31824929 10.3389/fbioe.2019.00312 PMC6882275

[8] Otto M, Wynands B, Marienhagen J, Blank LM, Wierckx N. Benzoate synthesis from glucose or glycerol using engineered *Pseudomonas taiwanensis*. Biotechnol J. 2020;15(11):e2000211. 32721071 10.1002/biot.202000211

[9] Wynands B, Kofler F, Sieberichs A, da Silva N, Wierckx N. Engineering a *Pseudomonas taiwanensis* 4-coumarate platform for production of *para*-hydroxy aromatics with high yield and specificity. Metab Eng. 2023;78:115–27. 37209862 10.1016/j.ymben.2023.05.004 PMC10360455

[10] Bitzenhofer NL, Kruse L, Thies S, Wynands B, Lechtenberg T, Rönitz J, et al. Towards robust *Pseudomonas* cell factories to harbour novel biosynthetic pathways. Essays Biochem. 2021;65(2):319–36. 34223620 10.1042/EBC20200173 PMC8314020

[11] Schwanemann T, Otto M, Wierckx N, Wynands B. *Pseudomonas* as versatile aromatics cell factory. Biotechnol J. 2020;15(11):e1900569. 32978889 10.1002/biot.201900569

[12] Meijnen JP, de Winde JH, Ruijssenaars HJ. Engineering *Pseudomonas putida* S12 for efficient utilization of d-xylose and l-arabinose. Appl Environ Microbiol. 2008;74(16):5031–7. 18586973 10.1128/AEM.00924-08 PMC2519266

[13] Meijnen JP, de Winde JH, Ruijssenaars HJ. Establishment of oxidative D-xylose metabolism in *Pseudomonas putida* S12. Appl Environ Microbiol. 2009;75(9):2784–91. 19270113 10.1128/AEM.02713-08 PMC2681702

[14] Le Meur S, Zinn M, Egli T, Thöny-Meyer L, Ren Q. Production of medium-chain-length polyhydroxyalkanoates by sequential feeding of xylose and octanoic acid in engineered *Pseudomonas putida* KT2440. BMC Biotechnol. 2012;12:53. 22913372 10.1186/1472-6750-12-53 PMC3542253

[15] Dvořák P, de Lorenzo V. Refactoring the upper sugar metabolism of *Pseudomonas putida* for co-utilization of cellobiose, xylose, and glucose. Metab Eng. 2018;48:94–108. 29864584 10.1016/j.ymben.2018.05.019

[16] Bator I, Wittgens A, Rosenau F, Tiso T, Blank LM. Comparison of three xylose pathways in *Pseudomonas putida* KT2440 for the synthesis of valuable products. Front Bioeng Biotechnol. 2019;7:480. 32010683 10.3389/fbioe.2019.00480 PMC6978631

[17] Wang Y, Horlamus F, Henkel M, Kovacic F, Schläfle S, Hausmann R, et al. Growth of engineered *Pseudomonas putida* KT2440 on glucose, xylose, and arabinose: hemicellulose hydrolysates and their major sugars as sustainable carbon sources. Glob Change Biol Bioenergy. 2019;11(1):249–59.

[18] Elmore JR, Dexter GN, Salvachúa D, O’Brien M, Klingeman DM, Gorday K, et al. Engineered *Pseudomonas putida* simultaneously catabolizes five major components of corn stover lignocellulose: glucose, xylose, arabinose, *p*-coumaric acid, and acetic acid. Metab Eng. 2020;62:62–71. 32828991 10.1016/j.ymben.2020.08.001

[19] Godoy P, García-Franco A, Recio MI, Ramos JL, Duque E. Synthesis of aromatic amino acids from 2G lignocellulosic substrates. Microb Biotechnol. 2021;14(5):1931–43. 34403199 10.1111/1751-7915.13844 PMC8449653

[20] Wu P, Ding H, Xu Z, Zhang Y, Dang Y, Gao B, et al. Engineering mixed sugar metabolic channels in *Pseudomonas putida* to produce vanillic acid. Synth Syst Biotechnol. 2026;11:277–86. 41141486 10.1016/j.synbio.2025.10.002 PMC12552152

[21] Köhler KA, Blank LM, Frick O, Schmid A. D-Xylose assimilation via the Weimberg pathway by solvent-tolerant *Pseudomonas taiwanensis* VLB120. Environ Microbiol. 2015;17(1):156–70. 24934825 10.1111/1462-2920.12537

[22] Nerke P, Korb J, Haala F, Hubmann G, Lütz S. Metabolic bottlenecks of *Pseudomonas taiwanensis* VLB120 during growth on D-xylose via the Weimberg pathway. Metab Eng Commun. 2024;18:e00241. 39021639 10.1016/j.mec.2024.e00241 PMC11252243

[23] Meijnen JP, Verhoef S, Briedjlal AA, de Winde JH, Ruijssenaars HJ. Improved *p*-hydroxybenzoate production by engineered *Pseudomonas putida* S12 by using a mixed-substrate feeding strategy. Appl Microbiol Biotechnol. 2011;90(3):885–93. 21287166 10.1007/s00253-011-3089-6 PMC3076579

[24] Ling C, Peabody GL, Salvachúa D, Kim YM, Kneucker CM, Calvey CH, et al. Muconic acid production from glucose and xylose in *Pseudomonas putida* via evolution and metabolic engineering. Nat Commun. 2022;13(1):4925. 35995792 10.1038/s41467-022-32296-y PMC9395534

[25] Rubinstein GM, Mokwatlo SC, Chirban L, Lepard AR, Ingraham MA, Ramirez KJ, et al. Integration of metabolic and bioprocess engineering for the production of β-ketoadipic acid from glucose and xylose by *Pseudomonas putida*. Green Chem. 2025;27(35):10673–85.

[26] Brack Y, Sun C, Yi D, Bornscheuer UT. Discovery of novel tyrosine ammonia lyases for the enzymatic synthesis of *p*-coumaric acid. ChemBioChem. 2022;23(10):e202200062. 35352477 10.1002/cbic.202200062 PMC9321829

[27] Ackermann YS, Li WJ, Op de Hipt L, Niehoff PJ, Casey W, Polen T, et al. Engineering adipic acid metabolism in *Pseudomonas putida*. Metab Eng. 2021;67:29–40. 33965615 10.1016/j.ymben.2021.05.001

[28] Op de Hipt L, Ackermann YS, de Jong H, Polen T, Wynands B, Wierckx N. Engineering of 1,4-butanediol and adipic acid metabolism in *Pseudomonas taiwanensis* for upcycling to aromatic compounds. Microb Biotechnol. 2025;18(8):e70205. 40788657 10.1111/1751-7915.70205 PMC12337976

[29] Johnson CW, Salvachúa D, Rorrer NA, Black BA, Vardon DR, St. John PC, et al. Innovative chemicals and materials from bacterial aromatic catabolic pathways. Joule. 2019;3(6):1523–37.

[30] Nikel PI, Kim J, de Lorenzo V. Metabolic and regulatory rearrangements underlying glycerol metabolism in *Pseudomonas putida* KT2440. Environ Microbiol. 2014;16(1):239–54. 23967821 10.1111/1462-2920.12224

[31] Espeso DR, Dvořák P, Aparicio T, de Lorenzo V. An automated DIY framework for experimental evolution of *Pseudomonas putida*. Microb Biotechnol. 2021;14(6):2679–85. 33047876 10.1111/1751-7915.13678 PMC8601172

[32] Dvořák P, Burýšková B, Popelářová B, Ebert BE, Botka T, Bujdoš D, et al. Synthetically-primed adaptation of *Pseudomonas putida* to a non-native substrate d-xylose. Nat Commun. 2024;15(1):2666. 38531855 10.1038/s41467-024-46812-9 PMC10965963

[33] Di Luccio E, Petschacher B, Voegtli J, Chou HT, Stahlberg H, Nidetzky B, et al. Structural and kinetic studies of induced fit in xylulose kinase from *Escherichia coli*. J Mol Biol. 2007;365(3):783–98. 17123542 10.1016/j.jmb.2006.10.068 PMC1995121

[34] Boulanger EF, Sabag-Daigle A, Thirugnanasambantham P, Gopalan V, Ahmer BMM. Sugar-phosphate toxicities. Microbiol Mol Biol Rev. 2021;85(4):e0012321. 34585982 10.1128/MMBR.00123-21 PMC8483676

[35] Nijland JG, Zhang X, Driessen AJM. D-xylose accelerated death of pentose metabolizing *Saccharomyces cerevisiae*. Biotechnol Biofuels Bioprod. 2023;16(1):67. 37069654 10.1186/s13068-023-02320-4 PMC10111712

[36] Woo S, Lim HG, Norton-Baker B, Lind TM, Gladden NE, Chen Y, et al. Simultaneous optimization of lignocellulosic sugar catabolism via systematic laboratory evolution in dynamic conditions. bioRxiv. 2026. 10.64898/2026.02.02.702459.42146489

[37] Wordofa GG, Kristensen M. Tolerance and metabolic response of *Pseudomonas taiwanensis* VLB120 towards biomass hydrolysate-derived inhibitors. Biotechnol Biofuels. 2018;11:199. 30034525 10.1186/s13068-018-1192-y PMC6052574

[38] Calero P, Jensen SI, Nielsen AT. Broad-host-range ProUSER vectors enable fast characterization of inducible promoters and optimization of *p*-coumaric acid production in *Pseudomonas putida* KT2440. ACS Synth Biol. 2016;5(7):741–53. 27092814 10.1021/acssynbio.6b00081

[39] Khlebnikov A, Risa O, Skaug T, Carrier TA, Keasling JD. Regulatable arabinose-inducible gene expression system with consistent control in all cells of a culture. J Bacteriol. 2000;182(24):7029–34. 11092865 10.1128/jb.182.24.7029-7034.2000 PMC94830

[40] Kolodrubetz D, Schleif R. Regulation of the l-arabinose transport operons in *Escherichia coli*. J Mol Biol. 1981;151(2):215–27. 6461771 10.1016/0022-2836(81)90512-x

[41] Schleif R. AraC protein, regulation of the l-arabinose operon in *Escherichia coli*, and the light switch mechanism of AraC action. FEMS Microbiol Rev. 2010;34(5):779–96. 20491933 10.1111/j.1574-6976.2010.00226.x

[42] Desai TA, Rao CV. Regulation of arabinose and xylose metabolism in *Escherichia coli*. Appl Environ Microbiol. 2010;76(5):1524–32. 20023096 10.1128/AEM.01970-09 PMC2832368

[43] Rönitz J, Herrmann F, Wynands B, Polen T, Wierckx N. SIGHT-A system for solvent-tight incubation and growth monitoring in high throughput. Eng Life Sci. 2025;25(2):e202400037. 39990769 10.1002/elsc.202400037 PMC11842283

[44] Chen X, Kuhn E, Jennings EW, Nelson R, Tao L, Zhang M, et al. DMR (deacetylation and mechanical refining) processing of corn stover achieves high monomeric sugar concentrations (230 g L^−1^) during enzymatic hydrolysis and high ethanol concentrations (>10% *v*/*v*) during fermentation without hydrolysate purification or concentration. Energy Environ Sci. 2016;9(4):1237–45.

[45] Wang Y, Zhang Y, Cui Q, Feng Y, Xuan J. Composition of lignocellulose hydrolysate in different biorefinery strategies: nutrients and inhibitors. Molecules. 2024;29(10):2275. 38792135 10.3390/molecules29102275 PMC11123716

[46] Kohlstedt M, Wittmann C. GC–MS-based ^13^C metabolic flux analysis resolves the parallel and cyclic glucose metabolism of *Pseudomonas putida* KT2440 and *Pseudomonas aeruginosa* PAO1. Metab Eng. 2019;54:35–53. 30831266 10.1016/j.ymben.2019.01.008

[47] Hartmans S, Smits JP, van der Werf MJ, Volkering F, de Bont JA. Metabolism of styrene oxide and 2-phenylethanol in the styrene-degrading *Xanthobacter* strain 124X. Appl Environ Microbiol. 1989;55(11):2850–5. 16348047 10.1128/aem.55.11.2850-2855.1989 PMC203180

[48] Puigbò P, Guzmán E, Romeu A, Garcia-Vallvé S. OPTIMIZER: a web server for optimizing the codon usage of DNA sequences. Nucleic Acids Res. 2007;35(Web Server issue):W126–31. 17439967 10.1093/nar/gkm219 PMC1933141

[49] Martínez-García E, de Lorenzo V. Engineering multiple genomic deletions in Gram-negative bacteria: analysis of the multi-resistant antibiotic profile of *Pseudomonas putida* KT2440. Environ Microbiol. 2011;13(10):2702–16. 21883790 10.1111/j.1462-2920.2011.02538.x

[50] Johnson CW, Beckham GT. Aromatic catabolic pathway selection for optimal production of pyruvate and lactate from lignin. Metab Eng. 2015;28:240–7. 25617773 10.1016/j.ymben.2015.01.005

[51] Chomczynski P, Rymaszewski M. Alkaline polyethylene glycol-based method for direct PCR from bacteria, eukaryotic tissue samples, and whole blood. Biotechniques. 2006;40(4):454–6, 8. 16629392 10.2144/000112149

[52] Ditta G, Stanfield S, Corbin D, Helinski DR. Broad host range DNA cloning system for Gram-negative bacteria: construction of a gene bank of *Rhizobium meliloti*. Proc Natl Acad Sci USA. 1980;77(12):7347–51. 7012838 10.1073/pnas.77.12.7347 PMC350500

[53] Choi KH, Gaynor JB, White KG, Lopez C, Bosio CM, Karkhoff-Schweizer RR, et al. A Tn7-based broad-range bacterial cloning and expression system. Nat Methods. 2005;2(6):443–8. 15908923 10.1038/nmeth765

[54] Kofler F, Schwanemann T, Teófilo da Silva N, Wierckx N, Wynands B. Engineering *Pseudomonas taiwanensis* for efficient chorismate-based production of mono- and dihydroxybenzoates. Metab Eng Commun. 2026;22:e00273. 41732675 10.1016/j.mec.2026.e00273 PMC12925505

[55] Ackermann YS, de Witt J, Mezzina MP, Schroth C, Polen T, Nikel PI, et al. Bio-upcycling of even and uneven medium-chain-length diols and dicarboxylates to polyhydroxyalkanoates using engineered *Pseudomonas putida*. Microb Cell Fact. 2024;23(1):54. 38365718 10.1186/s12934-024-02310-7 PMC10870600

[56] Abramson J, Adler J, Dunger J, Evans R, Green T, Pritzel A, et al. Accurate structure prediction of biomolecular interactions with AlphaFold 3. Nature. 2024;630(8016):493–500. 38718835 10.1038/s41586-024-07487-w PMC11168924

