# Supplementary material for: Expanding the substrate spectrum in engineered Pseudomonas taiwanensis for efficient production of 4-coumarate from lignocellulosic sugars
Source: Microb Cell Fact. 2026 May 23;25:130. doi: 10.1186/s12934-026-03014-w (PMC13200318; doi:10.1186/s12934-026-03014-w)
Supplement: Supplementary file 1 — Additional file 1. [file 12934_2026_3014_MOESM1_ESM.pdf]

Supplementary information to "Expanding the substrate spectrum in engineered *Pseudomonas taiwanensis* for efficient production of 4-coumarate from lignocellulosic sugars"

Benedikt Wynands[a], Sophia Feltes[a], Nadine Teófilo da Silva[a], Tino Polen[a], and Nick Wierckx[a]

[a]Institute of Bio- and Geosciences, IBG-1: Biotechnology, Forschungszentrum Jülich GmbH, 52425 Jülich, Germany

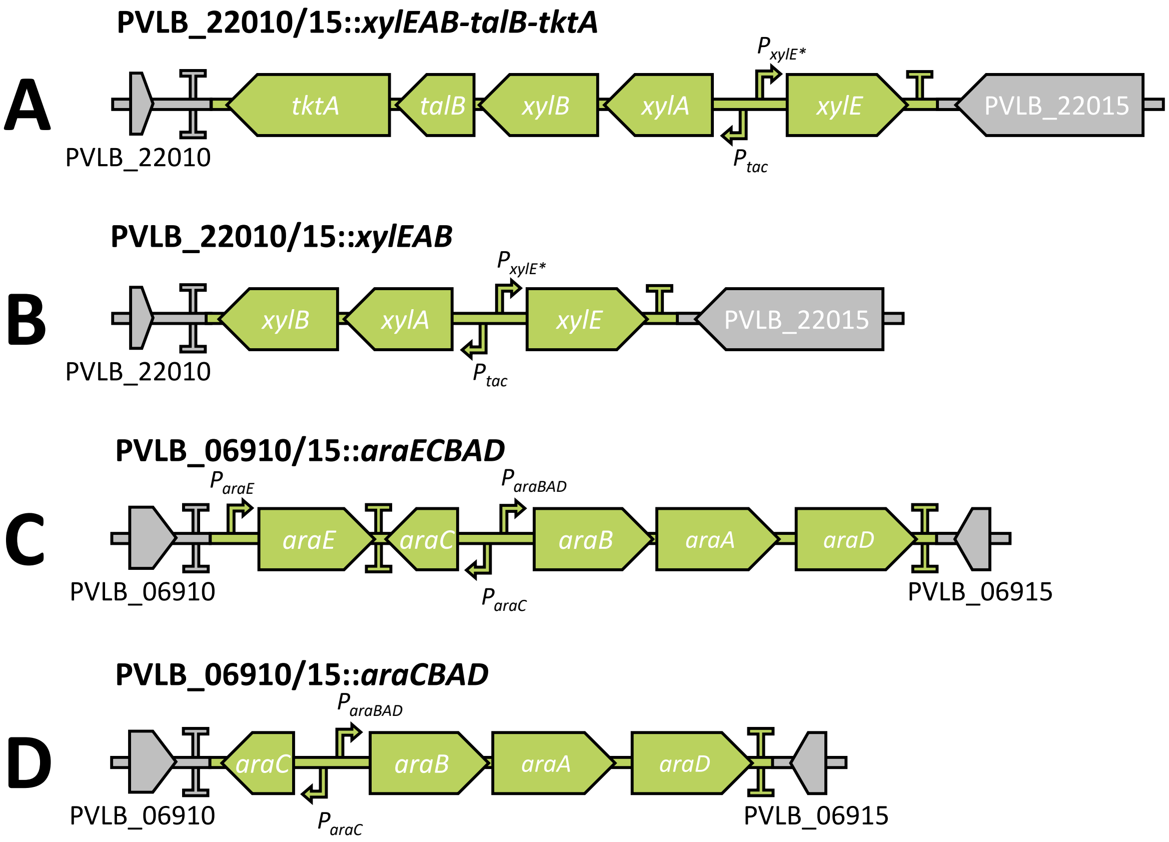

Figure S1 Genetic architecture of the integrated expression cassettes for the engineered metabolism of D-xylose (**A** and **B**) and L-arabinose (**C** and **D**). Light green colored parts indicate heterologous elements.

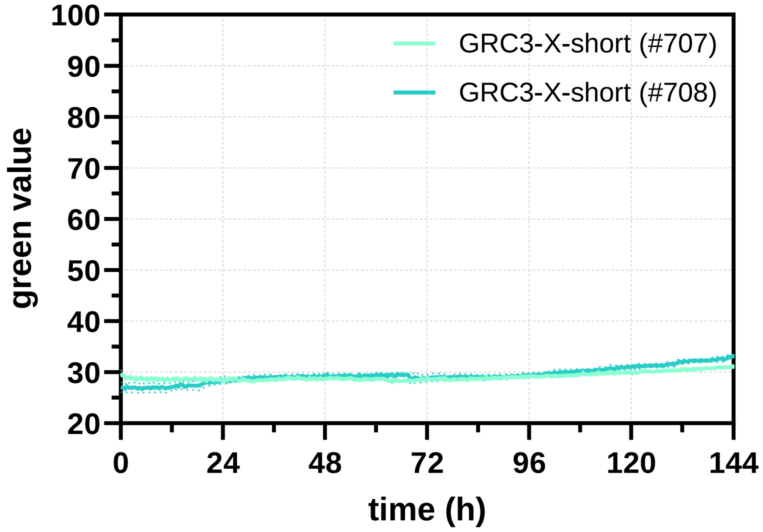

Figure S2 Growth analysis of two independent clones of strain GRC3-X-short (expressing *xylEAB* but lacking *talB-tktA*) in the Growth Profiler 960 using MSM with 24 mM xylose as sole carbon source. The lines represent means of four replicates (n = 4). The dotted lines indicate the standard deviation.

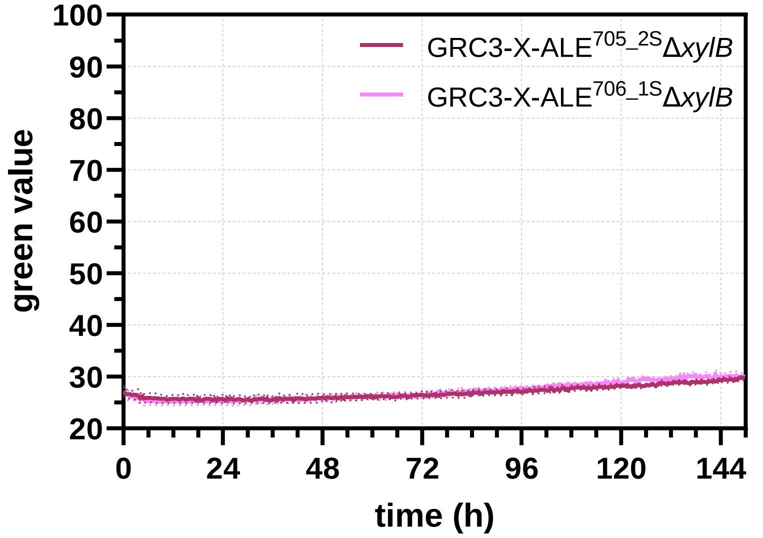

Figure S3 Growth analysis of GRC3-X-ALE^{705_2S}Δ*xylB* and GRC3-X-ALE^{706_1S}Δ*xylB* in the Growth Profiler 960 using MSM with 24 mM xylose as sole carbon source. The lines represent means of four replicates (n = 4). The dotted lines indicate the standard deviation.

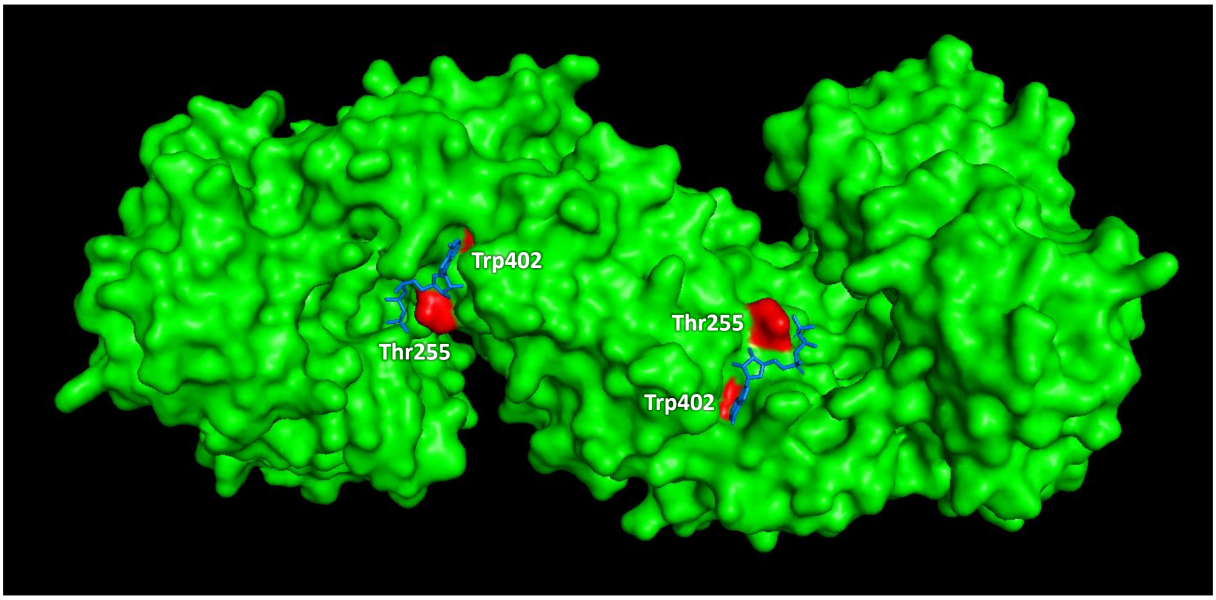

Figure S4 AlphaFold 3 prediction of the 3D structure of homodimeric xylulose kinase XylB from *Escherichia coli* with two ATP molecules (blue) and their suggested binding sites. The amino acid residues Thr255 and Trp402 that were mutated in evolved ALE strains are highlighted (red). Visualization was done using PyMOL.

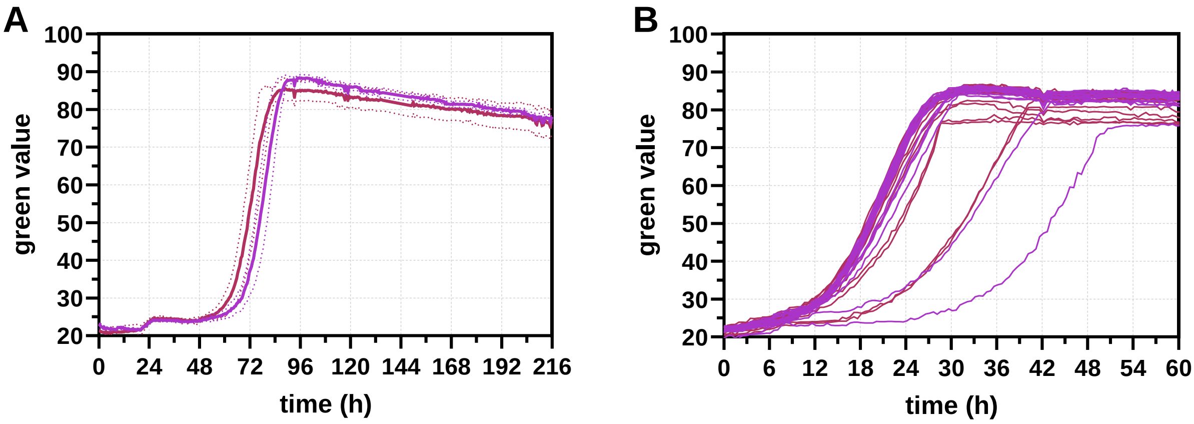

Figure S5 One-batch ALE for growth on arabinose of *P. taiwanensis* GRC3-A-REX^{705_2S} and growth of isolated clones derived from this ALE. (**A**) Arabinose ALE cultivation of two independent clones of strain GRC3-A-REX^{705_2S} (#2296 in pink and #2297 in maroon). Both clones were cultivated in three replicates in the Growth Profiler 960. The initial replicates were inoculated to an OD_{600} of 0.05 from one pre-culture. The lines represent means of replicates (n = 3). The dotted lines indicate the standard deviation. At the end of the cultivation, a small volume from each well was streaked onto LB agar to isolate single colonies. From each well six clones were selected for subsequent growth analysis. (**B**) Growth of the selected evolved clones in MSM with 24 mM arabinose. Dark pink lines represent clones that derived from strain GRC3-A-REX^{705_2S} clone #2296 and maroon pink ones those that derived from clone #2297. Four evolved clones were selected for WGS Pre-cultures were grown in MSM with 20 mM glucose, of which 2 µL were used to inoculate 198 µL medium of the main culture.

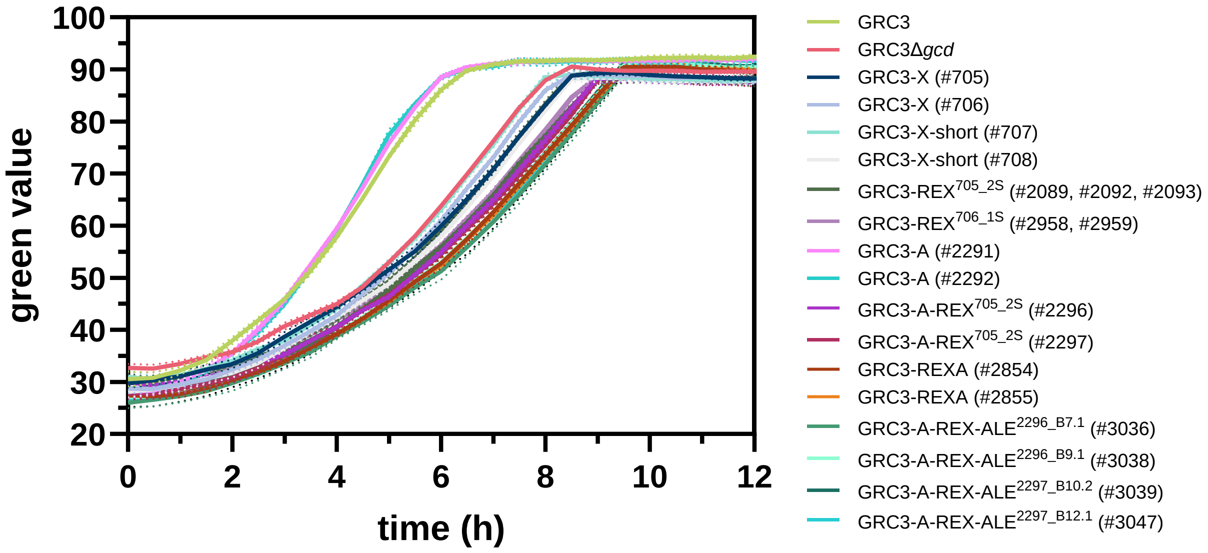

Figure S6 Growth kinetics of several engineered and evolved strains on glucose. The cultivation was performed in the Growth Profiler 960 using MSM with 20 mM glucose as sole carbon source. The lines represent means of four replicates (n = 4). The dotted lines indicate the standard deviation. Associated estimated growth rates can be found in Table S1.

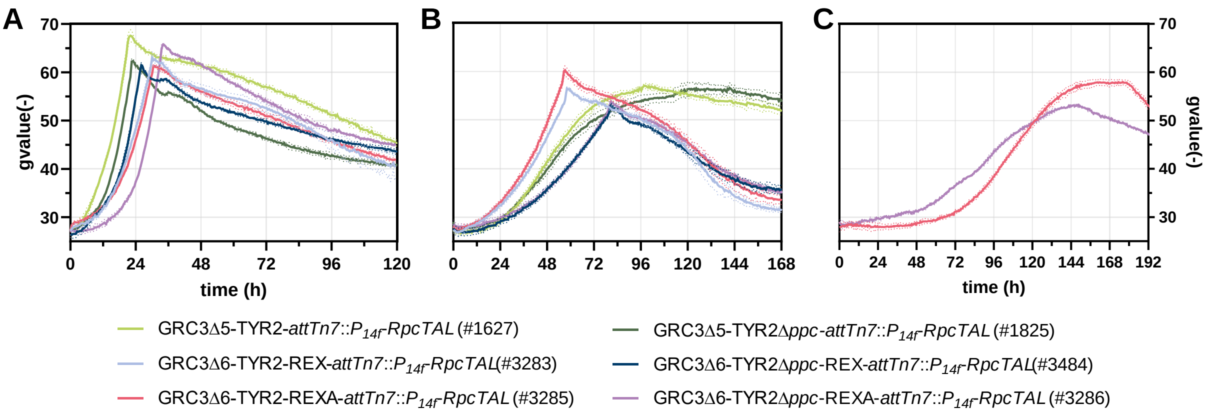

Figure S7 Growth of 4-coumarate producers on different lignocellulosic sugars. Growth was assessed in the Growth Profiler 960using two-fold-buffered MSM with 20 mM glucose after 120 h (**A**), 24 mM xylose after 168 h (**B**), and 24 mM arabinose after 192 h (**C**) as sole carbon source. Pre-cultures were grown with the same carbon source as the main culture in the Growth Profiler 960 using the SIGHT system [1]. The lines represent means of replicates (n = 4). The dotted lines indicate the standard deviation.

Table S1 Estimated growth rates from Growth Profiler cultivations.

Strain	Carbon source	Estimated growth rates (h^{-1})[a]	Fig.
GRC3	~20 mM glucose	0.59 ± 0.05	3A
GRC3Δ*gcd*	~20 mM glucose	0.39 ± 0.01	3A
GRC3-X (#705)	~20 mM glucose	0.37 ± 0.02	3A
GRC3-X (#706)	~20 mM glucose	0.45 ± 0.03	3A
GRC3	~24 mM xylose	0.29 ± 0.05<br>(early growth phase)	3B
GRC3-X (#705)	~24 mM xylose	0.16 ± 0.01<br>(late growth phase)	3B
GRC3-X (#706)	~24 mM xylose	0.19 ± 0.01<br>(late growth phase)	3B
GRC3	~20 mM glucose	0.64 ± 0.03	3C
GRC3Δ*gcd*	~20 mM glucose	0.46 ± 0.00	3C
GRC3-X-ALE^{705_2S} (#1800)	~20 mM glucose	0.38 ± 0.01	3C
GRC3-X-ALE^{706_1S} (#1801)	~20 mM glucose	0.40 ± 0.02	3C
GRC3-REX^{705_2S} (#2089)	~20 mM glucose	0.41 ± 0.02	3C
GRC3-REX^{705_2S} (#2092)	~20 mM glucose	0.40 ± 0.01	3C
GRC3-REX^{705_2S} (#2093)	~20 mM glucose	0.40 ± 0.01	3C
GRC3-REX^{706_1S} (#2958)	~20 mM glucose	0.42 ± 0.01	3C
GRC3-REX^{706_1S} (#2959)	~20 mM glucose	0.40 ± 0.01	3C
GRC3	~24 mM xylose	0.18 ± 0.01<br>(early growth phase)	3D
GRC3-X-ALE^{705_2S} (#1800)	~24 mM xylose	0.20 ± 0.01<br>(late growth phase)	3D
GRC3-X-ALE^{706_1S} (#1801)	~24 mM xylose	0.21 ± 0.00<br>(late growth phase)	3D
GRC3-REX^{705_2S} (#2089)	~24 mM xylose	0.23 ± 0.00<br>(late growth phase)	3D
GRC3-REX^{705_2S} (#2092)	~24 mM xylose	0.22 ± 0.00<br>(late growth phase)	3D
GRC3-REX^{705_2S} (#2093)	~24 mM xylose	0.23 ± 0.01<br>(late growth phase)	3D
GRC3-REX^{706_1S} (#2958)	~24 mM xylose	0.21 ± 0.00<br>(late growth phase)	3D
GRC3-REX^{706_1S} (#2959)	~24 mM xylose	0.21 ± 0.00<br>(late growth phase)	3D
GRC3-A-REX^{705_2S} (#2296)	~24 mM arabinose	0.15 ± 0.00	4A
GRC3-A-REX^{705_2S} (#2297)	~24 mM arabinose	0.16 ± 0.01	4A
GRC3-A-REX-ALE$^{2296_B7.1}$ (#3036)	~24 mM arabinose	0.19 ± 0.00	4B
GRC3-A-REX-ALE$^{2296_B9.1}$ (#3038)	~24 mM arabinose	0.20 ± 0.01	4B
GRC3-A-REX-ALE$^{2297_B10.2}$ (#3039)	~24 mM arabinose	0.19 ± 0.00	4B
GRC3-A-REX-ALE$^{2297_B12.1}$ (#3047)	~24 mM arabinose	0.19 ± 0.01	4B
GRC3-REXA (#2854)	~24 mM arabinose	0.20 ± 0.00	4B
GRC3-REXA (#2855)	~24 mM arabinose	0.20 ± 0.00	4B
GRC3	~20 mM glucose	0.57 ± 0.03	S6
GRC3Δ*gcd*	~20 mM glucose	0.40 ± 0.00	S6
GRC3-X (#705)	~20 mM glucose	0.40 ± 0.01	S6
GRC3-X (#706)	~20 mM glucose	0.45 ± 0.01	S6
GRC3-X-short (#707)	~20 mM glucose	0.44 ± 0.00	S6
GRC3-X-short (#708)	~20 mM glucose	0.43 ± 0.02	S6
GRC3-REX^{705_2S} (#2089)	~20 mM glucose	0.42 ± 0.02	S6
GRC3-REX^{705_2S} (#2092)	~20 mM glucose	0.42 ± 0.00	S6
GRC3-REX^{705_2S} (#2093)	~20 mM glucose	0.41 ± 0.02	S6
GRC3-REX^{706_1S} (#2958)	~20 mM glucose	0.43 ± 0.01	S6
GRC3-REX^{706_1S} (#2959)	~20 mM glucose	0.41 ± 0.01	S6
GRC3-A (#2291)	~20 mM glucose	0.65 ± 0.02	S6
GRC3-A (#2292)	~20 mM glucose	0.67 ± 0.02	S6
GRC3-A-REX (#2296)	~20 mM glucose	0.40 ± 0.01	S6
GRC3-A-REX (#2297)	~20 mM glucose	0.41 ± 0.02	S6
GRC3-A-REX-ALE$^{2296_B7.1}$ (#3036)	~20 mM glucose	0.41 ± 0.01	S6
GRC3-A-REX-ALE$^{2296_B9.1}$ (#3038)	~20 mM glucose	0.41 ± 0.00	S6
GRC3-A-REX-ALE$^{2297_B10.2}$ (#3039)	~20 mM glucose	0.41 ± 0.00	S6
GRC3-REXA (#2854)	~20 mM glucose	0.42 ± 0.00	S6
GRC3-REXA (#2855)	~20 mM glucose	0.40 ± 0.00	S6

[a] Growth rates were determined from OD_{600} equivalents as calculated from green values using a calibration.

Table S2 Mutation identified by whole-genome sequencing upon ALE on different carbon sources.

**Strain**	**Description**	**Mutations identified by WGS**
GRC3-X-ALE^{705_2S} (#1800)	clone 705_2S isolated from the sequential-batch ALE on xylose (culture for strain #705)	*xylB*^{W402R}
GRC3-X-ALE^{706_1S} (#1801)	clone 706_1S isolated from the sequential-batch ALE on xylose (culture for strain #706)	*xylB*^{T255A}
GRC3-A-REX-ALE$^{2296_B7.1}$ (#3036)	clone 2296_B7.1 isolated from one-batch ALE on arabinose (culture for strain #2296)	*araE*^{Q243*}
GRC3-A-REX-ALE$^{2296_B9.1}$ (#3038)	clone 2296_B9.1 isolated from one-batch ALE on arabinose (culture for strain #2296)	*araE*$^{\Delta 623bp}$ (frameshift)
GRC3-A-REX-ALE$^{2297_B10.2}$ (#3039)	clone 2297_B10.2 isolated from one-batch ALE on arabinose (culture for strain #2297)	*araE*^{Q243*}
GRC3-A-REX-ALE$^{2297_B12.1}$ (#3047)	clone 2297_B12.1 isolated from one-batch ALE on arabinose (culture for strain #2297)	*araE*^{Y136*}

Table S3 Plasmids used in this study.

Plasmid	Relevant characteristics	Reference
pEMG	Km^R, *oriV(R6K)*, *oriT*, *traJ*, *lacZα*-MCS flanked by two I-SceI restriction sites	Martínez-García and de Lorenzo [2]
pEMG-*ppc*	pEMG-derived plasmid with up- and downstream homology arms (TS1-*ppc*/TS2-*ppc*) of *ppc* for deletion of *ppc*	Wynands et al. [3]
pEMG-*gcd*	pEMG-derived plasmid with up- and downstream homology arms (TS1-*gcd*/TS2-*gcd*) of *gcd* for deletion of *gcd*	This study
pEMGg-*pheA*^{T310I}	pEMG-derived plasmid with P_{14f}→*msfgfp* and homology arms used for the implementation of the *pheA*^{T310I} mutation	Wynands et al. [4]
pK19*mobsacB*	Km^R, *oriV(colE1)*, *oriT*, *sacB*	Schäfer et al. [5]
pK19*mobsacB* -PVLB_22010/15-nox-xyl	pK19*mobsacB*-derived plasmid with homology arms flanking the expression construct *tktA-talB-xylBA*←P_{tac}-P_{xylE*}→*xylE* for chromosomal integration into the intergenic region of PVLB_22010 and PVLB_22015	This study
pK19*mob*sacB-PVLB_22010/15-nox-xyl-short	pK19*mobsacB*-derived plasmid with homology arms flanking the expression construct *xylBA*←P_{tac}-P_{xylE*}→*xylE* for chromosomal integration into the intergenic region of PVLB_22010 and PVLB_22015	This study
pK19*mobsacB* -PVLB_22010/15-nox-xyl(*xylB*^{W402R})	pK19*mobsacB*-derived plasmid with homology arms flanking the expression construct *tktA-talB-xylB*^{W402R}*A*←P_{tac}-P_{xylE*}→*xylE* for chromosomal integration into the intergenic region of PVLB_22010 and PVLB_22015	This study
pK19*mobsacB* -PVLB_22010/15-nox-xyl(*xylB*^{T255A})	pK19*mobsacB*-derived plasmid with homology arms flanking the expression construct *tktA-talB-xylB*^{T255A}*A*←P_{tac}-P_{xylE*}→*xylE* for chromosomal integration into the intergenic region of PVLB_22010 and PVLB_22015	This study
pSNW2-*xylB*	pSNW2-derived plasmid with up- and downstream homology arms (TS1-*xylB*/TS2-*xylB*) of *xylB* for deletion of *xylB*	This study
pBNW2	pSNW2-derived plasmid with *oriV(R6K)* replaced by *oriV(colE1)*	This study
pBNW2-PVLB_0690/15-*araECBAD*	pBNW2-derived plasmid with homology arms flanking the expression construct P_{araE}→*araE-araC*←P_{araC}-P_{araBAD}-*araBAD* for chromosomal integration into the intergenic region of PVLB_06910 and PVLB_06915	This study
pBNW2-PVLB_06910/15-*araCBAD*	pBNW2-derived plasmid with homology arms flanking the expression construct *araC*←P_{araC}-P_{araBAD}-*araBAD* for chromosomal integration into the intergenic region of PVLB_06910 and PVLB_06915	This study
pBG14f_*FRT_Kan*	Km^R flanked by FRT sites, *oriV(R6K)*, *oriT*, P_{14f}→*(BCD2)msfgfp*, mini-Tn7 transposon plasmid	Ackermann et al. [6]
pBG14f_*FRT_Kan_RtPAL*	pBG14f_*FRT_Kan*-derived plasmid for Tn7 transposition of the expression module P_{14f}→*(BCD2)RtPAL*	Wynands et al. [7]
pBG14f_*FRT_Kan_RpcTAL*	pBG14f_*FRT_Kan*-derived plasmid for Tn7 transposition of the expression module P_{14f}→*(BCD2)RpcTAL*	This study

Table S4 Cloning PCR primers used in this study.

Name	Sequence (5'→3')
BW097	ggataacagggtaatctgaattCGAATGCGCACCCGGGTC
BW098	tacggagctcGCACCACCGCTCCCCCAG
BW099	cggtggtgcgagctcCGTAGGTTCTCCGTTAGGTC
BW100	catgcctgcaggtcgactctagATCGCGCCGGGTACTACT
BW572	TAGAAAACCTCCTTAGCATG
BW575	GAATTCGAGCTCGGTACC
BW858	tgcatgcctgcaggtcgactACTTGGTCTCGTACGCAC
BW859	ccatttttttatggacgtcgGGTTCGCTGGCAATCAGTG
BW860	agccaaggagttgctgtgacGGCGATTGCAGAAGCAAATC
BW861	ggtacccggggatcctctagCGAAAAGTTTCACGCCAAG
BW881	CTCTACGCCGGACGCATC
BW882	GAATTAGCTTCACGCTGCC
BW883	cggcagcgtgaagctaattcGGCCGCGTTGCTGGCGTT
BW884	acgatgcgtccggcgtagagTCATGACCAAAATCCCTTAACGTGAGTTTTCGTTC
BW896	GTCGACTTAGGCCATCAGC
BW907	cgctgatggcctaagtcgacCGGCGATTGCAGAAGCAAATC
BW938	GGGGATCCTCTAGAGTCG
BW939	GGGTACCGAGCTCGAATTC
BW1254	tgaattcgagctcggtacccTGTTTGGTAGCGTGGACG
BW1255	gctggtacagGGACGTGCCCAAATCAATAC
BW1256	gggcacgtccCTGTACCAGCAGTTGCTG
BW1257	gtcgactctagaggatccccCCCTCTTTTTCCAGCTGTTC
BW1258	GTCGACAGGAGGTATAATATG
BW1259	AAATATCCTCCTCGTTGATC
BW1260	gatcaacgaggaggatatttATGTACATTGGTATTGATTTGGGCAC
BW1261	*atattatacctcctgtcgacTTAGGCCATCAGCGGCAG*
BW1324	taatctgaattcgagctcggtacccGGCTGGTACAGAAAGCCG
BW1328	agtcaaaagcctccgaccggaggcttttgactTCAAGCCGTCAATTGTCTG
BW1329	ttgcgcggtcccCTGAAAATCCATCAAAAAACCAG
BW1330	gatggattttcagGGGACCGCGCAAGCCTCT
BW1331	tgcaggtcgactctagaggatccccCTATGCTCTGAGGTGGCCGTTC
BW1350	cTGCAAAAGCGAAGATCCG
BW1351	cgctggcggatcttcgcttttgcaGCTGTAAAATTAGGTGGTTAATAATAATCTCAATAATTC
BW1352	agcctccggtcggaggcttttgacTCAGACGCCGATATTTCTC
BW1353	ggcggatcttcgcttttgcaTCAAGCCGTCAATTGTCTG
BW1354	TGCAAAAGCGAAGATCCG

Table S5 Cloning details on assembled plasmids.

pEMG-*gcd*	TS1-*gcd* and TS2-*gcd* were amplified from *P. taiwanensis* VLB120 gDNA with primers BW097/BW098 and BW099/BW100, respectively, using the Phusion High-Fidelity DNA Polymerase. pEMG was digested with EcoRI and XbaI, TS1-*gcd* with EcoRI and SacI, and TS2-*gcd* with SacI/SalI. Restriction digests were buffered using Tango buffer. The final plasmid was assembled by three-point ligation and transformed into electrocompetent *E. coli* DH5α λpir.
pK19*mobsacB*-PVLB_22010/15-nox-xyl	pK19*mobsacB* was linearized though PCR amplification with primers BW803/BW804 using the Q5 High-Fidelity 2X Master Mix. The homology arms TS1-PVLB_22010/15 and TS2-PVLB_22010/15 were amplified from *P. taiwanensis* VLB120 gDNA with the same DNA polymerase using primers BW858/BW859 and BW860/BW861. The expression cassette *xylEAB-talB-tktA* was obtained through the digestion of the plasmid pBxB1KanRxylose with XhoI and BamHI and gel extraction of the desired 7512-bp fragment. The plasmid was assembled by NEBuilder HiFi DNA Assembly and transformed into electrocompetent *E. coli* CopyCutter EPI400.
pK19*mob*sacB-PVLB_22010/15-nox-xyl-short	The fragment pK19*mobsacB*-PVLB_22010/15-*xylEAB* was amplified from pK19*mobsacB*-PVLB_22010/15-nox-xyl with primers BW907/BW896 using the Platinum SuperFi II DNA Polymerase to remove *talB-tktA*. Subsequently, the fragment was circularized by NEBuilder HiFi DNA Assembly and transformed into electrocompetent *E. coli* CopyCutter EPI400.
pK19*mobsacB* -PVLB_22010/15-nox-xyl(*xylB*^{W402R})	The fragment *talB-tktA*-pK19*mobsacB*-PVLB_22010/15-*xylEA* was amplified from pK19*mobsacB* -PVLB_22010/15-nox-xyl with primers BW1258/BW1259 using the Platinum SuperFi II Green PCR Master Mix. *xylB*^{W402R} was amplified from *P. taiwanensis* GRC3-X-ALE^{705_2S} gDNA with primers BW1260/BW1261 using the Q5 High-Fidelity 2X Master Mix. The plasmid was assembled by NEBuilder HiFi DNA Assembly and transformed into electrocompetent *E. coli* CopyCutter EPI400.
pK19*mobsacB* -PVLB_22010/15-nox-xyl(*xylB*^{T255A})	The fragment *talB-tktA*-pK19*mobsacB*-PVLB_22010/15-*xylEA* was amplified from pK19*mobsacB*-PVLB_22010/15-nox-xyl with primers BW1258/BW1259 using the Platinum SuperFi II Green PCR Master Mix. *xylB*^{T255A} was amplified from *P. taiwanensis* GRC3-X-ALE^{706_1S} gDNA with primers BW1260/BW1261 using the Q5 High-Fidelity 2X Master Mix. The plasmid was assembled by NEBuilder HiFi DNA Assembly and transformed into electrocompetent *E. coli* CopyCutter EPI400.
pSNW2-*xylB*	pSNW2 was linearized though PCR amplification with primers BW938/BW939 using the Q5 High-Fidelity 2X Master Mix. TS1-*xylB* and TS2-*xylB* were amplified from *P. taiwanensis* GRC3-X-ALE^{705_2S} gDNA with primers BW1254/BW1255 and BW1256/BW1257, respectively, using the Q5 High-Fidelity 2X Master Mix. The plasmid was assembled by NEBuilder HiFi DNA Assembly and transformed into chemically competent *E. coli* PIR2.
pBNW2	pSNW2 was linearized through PCR amplification with primers BW881/BW882 using the Q5 High-Fidelity 2X Master Mix. The *oriV(colE1)* was amplified from pGW26 with primers BW883/BW884 using the same DNA polymerase. The plasmid was assembled by NEBuilder HiFi DNA Assembly and transformed into chemically competent *E. coli* NEB 5-alpha.
pBNW2-PVLB_06910/15-*araECBAD*	pBNW2 was linearized through PCR amplification with primers BW938/BW939 using the Q5 High-Fidelity 2X Master Mix. The homology arm TS2-PVLB_06910/15 was amplified from *P. taiwanensis* VLB120 gDNA with the same DNA polymerase using primers BW1330/BW1331 while the homology arm TS1-PVLB_06910/15 was amplified from *P. taiwanensis* VLB120 gDNA with the Platinum SuperFi II Green PCR Master Mix using primers BW1324/BW1350. *araE* and *araCBAD* were amplified from *E. coli* K-12 MG1655 gDNA with primers BW1351/BW1352 and BW1328/BW1329, respectively, using the Platinum SuperFi II Green PCR Master Mix. The plasmid was assembled by NEBuilder HiFi DNA Assembly and transformed into chemically competent *E. coli* NEB 5-alpha.
pBNW2-PVLB_06910/15-*araCBAD*	The fragment *araCBAD*-pBNW2-PVLB_06910/15 was amplified with primers BW1353/BW1354 using the Platinum SuperFi II Green PCR Master Mix. Subsequently, the fragment was circularized by NEBuilder HiFi DNA Assembly and transformed into chemically competent *E. coli* NEB 5-alpha.
pBG14f_*FRT_Kan_RpcTAL*	The pBG14f_*FRT_Kan* backbone was amplified with primers BW575/BW572 using the Q5 High-Fidelity 2X Master Mix. *RpcTAL* was ordered as synthetic DNA fragment equipped with the required overhangs. NEBuilder HiFi DNA Assembly was used to assemble the plasmid that was transformed into chemically competent *E. coli* PIR2.

Codon-optimized *RpcTAL* coding sequence (5’→3’):

```
ATGAATACCGTTCGTCTGACGAAGAACACCGTAAGCCAAAAGAGCTTCAGCTTTCTCAACAACTCCGA
CGCGTCCGTGATCGTGGGTGATCGCCAGCTCACCATCGAAGAGGTCGTAAGCGTAGCGCGTTACCGGG
CCCGCGTGAAGCTGACGGAGAACCTGGAGAAGCTCGCTAACGTCCAAGCCAGCTGCGACTTCATCCGC
GATGCCGTCGAGTCCGGCGAACCGATCTACGGCGTCACCACCGGCTTCGGCGGTATGGCCAACGTCGT
GATCAGCCCAGAGAGCGCCACCTTGCTGCAAAACAACCTGATGTGTTACCATAAGGTCGGCGCCGGCA
ACAAGCTGCCACTCGCCGACGTACGCGCTGCCATGCTGCTGCGTGCTAACAGTCACGTCGCCGGGGCG
AGCGGCATCCGCCTGGAGCTGATCAAGCGTATGCTGATTTTCTTGAACGCTGGTGTGACCCCGCACGT
GCCGGAGTTCGGCAGCATCGGCGCATCGGGCGACCTGACCCCGCTGGCGTACATCTCGGGTGCCTTGA
TTGGGCTCAATAGCAGCTATATCGTGGACTTCGACGGTGAGGAGATGGATGCCCCCACCGCCTTGCAG
AAGCTGGGCCTGGAGCCCCTGCAACTGCTGCCTAAGGAAGGTCTGGCCATGATGAATGGTACTAGCGT
TATGACGGGCATCGCTGCCAATTGCGTACAGGATACCCGCATCCTCCTGGCTCTGAGCGTGGCGACCC
ATGCCCTGACCATCCAGGGCCTCGAGGGCACGAATCAGAGCTTCCACGAGTATATCCATAAACTGAAG
CCGCATAGCGGTCAGATCTGGGCCGCAAGCCAGATGCTGGAGCTGCTGGCTGGTAGCGGTCTGATCCG
GGACGAATTGGATGGCAGCCATGACTACCGGGGTAAAAACCCGATCCAGGATCGTTACAGCTTGCGTT
GCCTGCCTCAGTACATGGGCCCTATCGTAGACGGTATCGAAGACATCGCCAAGCAAGTGGAGATCGAA
ATCAACTCGGTGACCGACAACCCACTCATCGACGTGGAGAACCAAGCGTCCTACCATGGCGGGAACTT
CCTGGGCCAGTACATCGGCGTGGGCATGGACCGCCTGCGTTACCATATCGGTATGCTGGCCAAACATC
TGGACGTACAAATCGCGTACCTGGTGGCCCCCGAGTTTAACAACGGGCTGTCGCCGTCGCTGGTGGGC
AACCAGCAGCGCACGGTGAACATGGGGCTGAAAGGCCTGCAAATCACCGGCAACTCGATTATGCCGCT
GCTGACCTTCTACGGGAACTCGATCGCCGACCGCTTCCCGACCCACGCGGAGCAGTACAATCAGAACA
TCAACTCGCAGGGTTTCGCCTCCGCAAACCTGGCCCGTACCTCCGTAGAGATCTTCCAGCAGTATATT
GCGCTGGCACTGATGTTCGGCGTCCAGAGTGTGGACCTGCGCACCTACGCCATTGCCGGGCATTATGA
TGCGCGCGCCACCCTGTCCCCCGCCACGCAAGATCTGTACATGGCCGTTCGCAACGTGGTAGGCCGCC
CTCCAAGCAAGGAACGCGCCTACATCTGGGATGACAACGAGCAGGGCCTCGACAGCCACATCAGTAAG
ATCGCCGATGACATTGCCTATGGCGGCCAGATCGTGACCGCGATCTCCGAGGTGTTGTCGGCCCTGAA
GAGCGTGAATAACTAA
```

References

1. Rönitz J, Herrmann F, Wynands B, Polen T, Wierckx N. SIGHT-A system for solvent-tight incubation and growth monitoring in high throughput. Eng Life Sci. 2025;25(2):e202400037.
2. Martínez-García E, de Lorenzo V. Engineering multiple genomic deletions in Gram-negative bacteria: analysis of the multi-resistant antibiotic profile of *Pseudomonas putida* KT2440. Environ Microbiol. 2011;13(10):2702-16.
3. Wynands B, Otto M, Runge N, Preckel S, Polen T, Blank LM, et al. Streamlined *Pseudomonas taiwanensis* VLB120 chassis strains with improved bioprocess features. ACS Synth Biol. 2019;8(9):2036-50.
4. Wynands B, Lenzen C, Otto M, Koch F, Blank LM, Wierckx N. Metabolic engineering of *Pseudomonas taiwanensis* VLB120 with minimal genomic modifications for high-yield phenol production. Metab Eng. 2018;47:121-33.
5. Schäfer A, Tauch A, Jäger W, Kalinowski J, Thierbach G, Pühler A. Small mobilizable multi-purpose cloning vectors derived from the *Escherichia coli* plasmids pK18 and pK19: selection of defined deletions in the chromosome of *Corynebacterium glutamicum*. Gene. 1994;145(1):69-73.
6. Ackermann YS, Li WJ, Op de Hipt L, Niehoff PJ, Casey W, Polen T, et al. Engineering adipic acid metabolism in *Pseudomonas putida*. Metab Eng. 2021;67:29-40.
7. Wynands B, Kofler F, Sieberichs A, da Silva N, Wierckx N. Engineering a *Pseudomonas taiwanensis* 4-coumarate platform for production of *para*-hydroxy aromatics with high yield and specificity. Metab Eng. 2023;78:115-27.
